# Targeting Epigenetics in Ischemic Stroke: From Mechanisms to Therapeutics

**DOI:** 10.2174/011570159X462267260120104242

**Published:** 2026-04-09

**Authors:** Xiao Lan, Xin Xie, ChangQing Deng

**Affiliations:** 1 College of Integrated Chinese and Western Medicine, Key Laboratory of Hunan Provincial for Integrated Traditional Chinese and Western Medicine on Prevention and Treatment of Cardio-Cerebral Diseases, Hunan University of Chinese Medicine, Hunan, Changsha, 410208, China

**Keywords:** Ischemic stroke, epigenetics, DNA methylation, histone modification, miRNA, LncRNA, therapeutic targets

## Abstract

Ischemic stroke is the main cause of death and disability in human diseases worldwide. Due to the complexity of its pathophysiological mechanism, the treatment effect is not ideal. Epigenetics refers to the mechanisms of affecting gene function and expression by chemical modification or chromatin structure without changing the DNA sequence. The epigenetic network is mainly composed of various epigenetic modifying enzymes (such as DNA methyltransferase/demethylase, DNMT/tet, histone methyltransferase/demethylase, HMT/hdm, histone acetyltransferase/deacetylase hat/hdac) and non-coding RNAs with different functions. Epigenetic enzymes or non-coding RNAs can participate in the core pathophysiological processes after ischemic stroke, such as neuroinflammation, oxidative stress, and cell apoptosis, by regulating the activation or silencing of key gene transcription. Due to the regulatory role of epigenetic modifying enzymes and non-coding RNAs after ischemic stroke, targeted intervention of these epigenetic modifying enzymes and non-coding RNAs has been confirmed to improve the pathological process and neurological outcome after ischemia. Therefore, they are regarded as a series of therapeutic targets with great research and development potential. This study systematically reviewed the role and mechanism of DNA methylation, histone modification, and non-coding RNA in the pathological development of ischemic stroke, and discussed the research progress of related therapeutic drugs targeting these epigenetic modifying enzymes, in order to provide a theoretical basis and ideas for the development of new clinical diagnosis and treatment strategies for ischemic stroke.

## 1 INTRODUCTION

Stroke is a leading global cause of disability and mortality. According to the latest statistics from the American Heart Association, the global number of stroke patients reached 93.82 million in 2021, among which ischemic stroke (69.94 million cases) is the predominant subtype, accounting for 74.5% of all stroke cases [1]. Ischemic Stroke (IS) refers to a clinical syndrome characterized by rapid neurological deficits caused by various factors such as cerebral blood supply disorders and local cerebral ischemia, hypoxia, and necrosis. Current clinical treatments mainly rely on reperfusion therapies with strict time windows, but they have limitations, including a narrow therapeutic window, high risk of reperfusion injury, and inability to effectively intervene in post-ischemic cascade reactions. Therefore, breaking through the existing therapeutic framework and revealing new dimensions of the pathophysiological mechanisms of ischemic stroke have become key scientific issues urgently needing to be solved in this field.

Epigenetics is a heritable and modifiable process that regulates gene expression without changing the DNA sequence. In recent years, the role of epigenetic regulation in ischemic stroke has attracted increasing attention. Through multiple mechanisms such as DNA methylation, histone modifications, and non-coding RNAs, it dynamically and reversibly regulates gene expression, thereby extensively participating in core links of the ischemic cascade reaction, including neuroinflammation, oxidative stress, cell apoptosis, and blood-brain barrier disruption. Compared with traditional therapies, epigenetic interventions have the advantages of multi-targeting, reversibility, and a potential broad time window for treatment, and are expected to provide new strategies from neuroprotection to neurorepair for ischemic stroke. However, there is still a lack of systematic analysis of the temporal regulation, interaction networks, and translational potential of different epigenetic mechanisms in the ischemic cascade, which limits understanding of overall progress in this field and the clarity of clinical translation pathways [2, 3]. This article systematically reviews the roles of three key epigenetic mechanisms: DNA methylation, histone modifications (focusing on methylation and acetylation), and non-coding RNAs (miRNA and lncRNA) in the pathophysiological process of ischemic stroke, comments on the potential therapeutic drugs targeting these mechanisms and research progress, and discusses the challenges and prospects of their clinical translation. By constructing an integrated framework of "epigenetic ischemic cascade reaction", this article provides a new perspective for an in-depth understanding of the molecular mechanisms of ischemic stroke and theoretical basis and ideas for the development of new therapeutic strategies.

## 2 EPIGENETIC MECHANISMS IN ISCHEMIC STROKE

In the process of epigenetic modification, DNA modification and histone modification have specific epigenetic "writers", "readers," and "erasers". "Readers" have unique domains that can specifically recognize and bind to methylated sites on DNA or methylated/acetylated modification sites on histones. For example, the Methyl CpG binding domain (MBD) protein family can recognize DNA methylation sites, while Ubiquitin-like with PHD and RING finger domains 1 (UHRF1) can target hemimethylated DNA sites. Other "readers" include the Plant Homeodomain (PHD) that can recognize histone methylation and acetylation modifications. By recognizing these modifications, "readers" act as "decoders" of DNA and histone codes to regulate downstream gene expression. "Writers" include enzymes that catalyze the addition of epigenetic marks: DNA methyltransferases (DNMTs) are responsible for DNA methylation modification, histone acetyltransferases (HATs) catalyze histone acetylation; in addition, histone methyltransferases (HMTs) can mediate histone methylation, acting on lysine, arginine, and histidine residues. On the contrary, "erasers" are enzymes that remove these epigenetic marks: Ten Eleven Translocation (TET) proteins are involved in the process of DNA demethylation, and histone deacetylases (HDACs) catalyze histone deacetylation.

The coordinated action of "writers", "readers", and "erasers" regulates gene promoter regions by forming activating epigenetic marks (such as H3K4me3, H3K36me3, H3K79me3) or inhibitory epigenetic marks (such as H3K9me3, H3K27me3, H4K20me3), thereby affecting gene transcription activity and realizing precise regulation of gene expression (Fig. **1**) [4].

### 2.1 DNA Methylation and Demethylation

In eukaryotic cells, DNA methylation, that is, the addition of a methyl group to the 5th carbon atom of cytosine (forming 5-methylcytosine, 5mC), is a key epigenetic modification involved in chromatin remodeling and gene regulation processes. This modification mainly occurs at cytosine-guanine (CpG) dinucleotide sites. Methylation of CpG islands in gene promoter regions is catalyzed by DNMTs, including DNMT1, DNMT3A, and DNMT3B, and is closely related to transcriptional inhibition [5]. This inhibitory effect is mediated through two main mechanisms: (1) directly hindering the binding of transcription factors to gene promoters or other cis-regulatory elements, and (2) indirectly promoting gene silencing by recruiting transcriptional repressor complexes through MBD proteins [6].

DNA demethylation can be divided into passive demethylation and active demethylation. Passive demethylation refers to the gradual dilution of methylated sites during the DNA replication cycle, while active demethylation is a process catalyzed by demethylases independent of DNA replication. The active demethylation pathway is mainly mediated by the TET protein family [7].

The dynamic balance of DNA methylation mediated by DNMTs and TET proteins is an important prerequisite for maintaining normal gene expression, and the imbalance of this balance is the pathological basis for the occurrence of various diseases.

#### 2.1.1 DNMT-Mediated DNA Methylation

##### 2.1.1.1 DNMT1

In the mouse photothrombotic stroke (PTS) model, DNMT1 expression is significantly upregulated, and inhibiting its expression can reduce neuronal apoptosis [8]. at the same time, DNMT1 upregulation can promote hypermethylation of the CpG island in the promoter region of tissue inhibitor of metalloproteinases 2 (TIMP2), inhibit TIMP2 expression, lead to imbalance of the matrix metalloproteinase 9 (MMP 9)/TIMP 2 balance, increase MMP 9 activity, thereby degrade the extracellular matrix and destroy the blood brain barrier, and aggravate cerebral infarction [9]. The DNMT1 inhibitor 5-azacytidine can reverse TIMP2 methylation and maintain barrier integrity [10].

In addition, DNMT1 can recruit Methyl CpG binding domain 2 (MeCP2) and repressor element-1 silencing transcription factor (REST) to form a transcriptional repressor complex, leading to hypermethylation of the sodium calcium exchanger 1 (NCX1) promoter region and downregulation of its expression, thereby aggravating injury (11); knockout of DNMT1, MeCP2 or REST can restore NCX1 expression, and the protective effect of 5 azacytidine disappears after NCX1 knockout, confirming that NCX1 is a key downstream effector molecule of this pathway [11].

These research results indicate that DNMT1, as an upstream molecular switch, can simultaneously activate damaging pathways and inhibit protective pathways. Therefore, DNMT1 may become an important therapeutic target for improving the prognosis of ischemic stroke.

##### 2.1.1.2 DNMT3A and DNMT3B

After ischemic stroke, DNMT3A expression is upregulated, and silencing or inhibiting DNMT3A can reduce post-ischemic neuronal apoptosis [12, 13]. It is worth noting that Lyu *et al*. reported opposite results: in the mouse transient Middle Cerebral Artery Occlusion (tMCAO) model, inhibiting DNMT3A with RG108 can aggravate neutrophil infiltration and neuroinflammation, leading to an increase in cerebral infarction volume [14]. The discrepancy may arise from the factors such as differences in the chemical nature of the inhibitors. Previous studies utilized 5-Azacytidine, a pan-DNMT inhibitor (primarily affecting DNMT1), whose neuroprotective effects may have obscured the function of DNMT3A. In contrast, RG108 exhibits relative selectivity for DNMT3A, leading to divergent outcomes. Second, Lyu *et al*. [14] administered RG108 intravenously, which likely failed to cross the blood-brain barrier at effective concentrations to exert central inhibition of DNMT3A. Instead, their findings may reflect the role of DNMT3A in peripheral immune cells, specifically, in maintaining immune homeostasis. Inhibiting peripheral DNMT3A could thus trigger a systemic pro-inflammatory response, exacerbating cerebral injury. Third, DNMT3A may serve distinct functions in different cell types. In neuronal cells, it may predominantly suppress the expression of "injury"-related genes, whereas in circulating neutrophils, its function may involve restraining excessive activation. Future research should focus on elucidating the specific functions and molecular mechanisms of DNMT3A in different cell types, particularly neurons and peripheral immune cells. Building on this understanding, targeted intervention strategies with cell selectivity or microenvironment-responsive properties should be developed to overcome the therapeutic contradictions posed by its "context-dependent" nature, thereby paving new pathways for precision epigenetic therapy in ischemic stroke.

DNMT3B expression is also increased after ischemic stroke. Downregulating DNMT3B through gene knockout, drug inhibition (SGI1027), or exosomal miRNA delivery can promote demethylation of the promoter regions of neuroprotective genes such as *PINK1* [15], *MAP4K4* [16], and *PI3K* [17]. These findings suggest that DNMT3B participates in the pathological process of ischemic brain injury by simultaneously silencing multiple protective pathways.

In summary, members of the DNMT family are abnormally upregulated and involved in the pathological process of ischemic stroke, and aggravate cerebral infarction by inhibiting protective pathways and activating damaging pathways. Intervening in their mechanisms of action may be an important target for improving the prognosis of ischemic stroke.

#### 2.1.2 TET-Mediated DNA Demethylation

The TET protein family belongs to the α-ketoglutarate/ Fe^2+^-dependent dioxygenase superfamily. The currently recognized demethylation pathway is as follows: TET enzymes first oxidize 5mC to 5-hydroxymethylcytosine (5-hydroxymethylcytosine, 5hmC); subsequently, 5hmC is further oxidized to 5-formylcytosine (5-formylcytosine, 5fC) and 5-carboxylcytosine (5-carboxylcytosine, 5caC); Thymine DNA glycosylase (TDG) then excises 5caC, ultimately restoring unmodified cytosine to achieve active DNA demethylation. TET proteins are highly expressed in the brain and serve as core enzymes in the DNA demethylation process [18].

##### 2.1.2.1 TET1

In the rat Middle Cerebral Artery Occlusion (MCAO) model and Oxygen-Glucose Deprivation (OGD)-treated astrocytes, TET1 expression is significantly downregulated after ischemia. Overexpression of TET1 can upregulate oxygen-regulated protein 150 (ORP150), thereby alleviating endoplasmic reticulum stress, maintaining calcium homeostasis, and inhibiting astrocyte apoptosis [19].

Notably, TET1 expression in microglia increases with prolonged reperfusion time. TET1 can reduce the RNA m^5^C modification of RelB (this modification decreases RelB mRNA stability), thereby enhancing RelB RNA stability and protein expression and exacerbating neuroinflammation [20].

This contradiction highlights the cell type specificity and pathological stage dependency of epigenetic regulation. Future research needs to systematically analyze the dynamic expression patterns and functional differences of DNMT3A in different neural cell types (such as astrocytes, microglia, neurons) and at different pathological stages. Additionally, it is essential to elucidate the epigenetic mechanisms through which DNMT3A regulates downstream target genes (such as inflammatory factors and endoplasmic reticulum stress-related proteins) in specific cellular environments. This will provide a theoretical basis for developing precision epigenetic intervention strategies tailored to specific cell types and disease stages.

##### 2.1.2.2 TET2

In the brain tissue of the mouse Middle Cerebral Artery Occlusion/Reperfusion (MCAO/R) model, both 5hmC levels and TET2 expression are upregulated. Inhibiting TET2 expression reduces 5hmC abundance and aggravates cerebral infarction. The underlying mechanism is that TET2 promotes the conversion of 5mC to 5hmC in the promoter region of Brain-Derived Neurotrophic Factor (BDNF), enhancing BDNF transcription [21].

Additionally, TET2 can recruit histone deacetylase 2 (HDAC2) to the interleukin 6 (IL-6) promoter region, inhibiting its transcription through histone deacetylation [22]. In a neonatal hypoxic-ischemic model, TET2 recruits HDAC3 to inhibit p65 acetylation; knockout of TET2 enhances p65 acetylation and the expression of proinflammatory factors, exacerbating neuroinflammation [23].

Regarding vascular effects, TET2 expression in vascular endothelial cells is downregulated under hypoxia. Knockout of TET2 aggravates vascular endothelial injury through molecular mechanisms, including TET2-mediated demethylation and transcriptional activation of Signal Transducer and Activator of Transcription 3 (STAT3) (the protective effect disappears after STAT3 knockdown) [24], and downregulation of fructose-1,6-bisphosphatase 1 (FBP1) to promote glycolytic reprogramming [25].

These results indicate that TET2 may be a key target for improving the prognosis of ischemic stroke. On one hand, TET2 can alleviate ischemia-induced neuroinflammation and brain injury by inhibiting nuclear factor κB (NF-κB) p65 activation, blocking microglial M1 polarization, and reducing proinflammatory factor release. On the other hand, TET2 exerts neuroprotective effects by promoting BDNF transcription and enhancing angiogenesis. However, most current studies on TET2-mediated angiogenesis are *in vitro* endothelial cell experiments, and its role and regulatory mechanisms in *in vivo* animal experiments require further verification.

##### 2.1.2.3 TET3

TET3 expression is rapidly upregulated in the ischemic penumbra. Knockdown of TET3 aggravates brain injury, while enhancing its activity with L-ascorbic acid reduces cerebral infarction [26]. The potential mechanism is that TET3 promotes 5 hmC in the promoter regions of neuroprotective-related genes (such as BDNF, Vascular Endothelial Growth Factor (VEGF), forkhead box O (FOXO) family genes, and Wnt pathway genes), thereby enhancing their transcriptional expression and exerting protective effects [27].

Notably, the neuron-specific TET3 isoform lacks a DNA-binding domain and cannot directly bind to DNA to catalyze the conversion of 5mC to 5hmC [28]. It needs to form a complex with TIR domain adaptor protein (TILR) to initiate catalytic function, and knockdown of TILR completely abolishes the neuroprotective effect of TET3 [29]. These results demonstrate that TET3 functions by regulating the DNA hydroxymethylation of neuroprotective genes, and its interaction with TILR is critical for functional activation in neurons, providing theoretical support for targeted precision epigenetic therapy against TET3.

In summary, DNA methyltransferases (DNMTs) and TET enzymes collectively shape the pathological microenvironment of ischemic stroke by regulating DNA methylation dynamics. Targeting the subtype-specific functions of these enzymes may provide a key breakthrough for epigenetic therapy of ischemic stroke. Future research can be carried out in two directions: first, further explore the "epigenetic transcriptome" network regulated by DNMTs and TET after ischemic stroke to fully clarify the epigenetic and pathological mechanisms of DNA methylation in ischemic stroke; second, actively develop specific intervention strategies targeting DNMT expression or activity and their related transcriptional repressor complexes, which is expected to open up new epigenetic therapeutic approaches for ischemic stroke. The epigenetic regulatory mechanisms of different DNMT and TET isoforms in ischemic stroke are systematically summarized in Table **1**.

### 2.2 Histone Methylation and Demethylation

The basic structural unit of eukaryotic chromatin is the nucleosome, whose core consists of a histone octamer (containing two molecules each of H2A, H2B, H3, and H4), with DNA wrapped around the core approximately 1.75 turns. The N-terminal tails of core histones can undergo various post-translational modifications such as methylation, acetylation, and ubiquitination. These modifications precisely regulate transcriptional activity by altering chromatin structure or recruiting specific effector proteins [31].

Histone methylation is one of the key regulatory mechanisms in epigenetics, extensively participating in various pathophysiological processes by influencing chromatin structure, genomic stability, and gene transcriptional activity [32]. Histone methyltransferases and demethylases regulate gene transcriptional silencing and activation by modifying specific residues (*e.g*., H3K9, H3K27), ultimately converging on the modulation of core pathological modules such as cell death, inflammatory responses, and neural plasticity. The following sections will systematically elaborate on the histone methyltransferases and demethylases that have been extensively studied in ischemic stroke research.

#### 2.2.1 Histone Methyltransferases

In mammals, histone lysine methyltransferases include the SUV, EZH, SMYD, and SETD families. These enzymes precisely regulate key biological processes such as gene transcription and chromatin state maintenance by specifically catalyzing methylation at different sites and to specific degrees. The following focuses on histone lysine methyltransferases associated with ischemic stroke in recent studies.

##### 2.2.1.1 SUV39H1 and G9a

Both SUV39H1 and G9a (also known as EHMT2) belong to the SUV family of histone methyltransferases [33].

In the mouse photothrombotic stroke (PTS) model, the expression of SUV39H1 and G9a is significantly upregulated. Inhibition of G9a with the specific inhibitor A-366, or dual inhibition of G9a/GLP with BIX01294, can reduce neuronal apoptosis in the ischemic penumbra and narrow the infarct volume [30]. Further verification in the rat primary cortical neuron Oxygen-Glucose Deprivation/Reperfusion (OGD/R) model shows that inhibiting SUV39H1 and G9a can increase the acetylation level of H3K9 in the BDNF promoter region, enhance BDNF mRNA transcription, and thereby inhibit neuronal apoptosis [34].

Studies have demonstrated that inhibiting the abnormal activation of SUV39H1 and G9a after ischemia exerts neuroprotective effects by alleviating H3K9 methylation-mediated transcriptional repression and promoting the expression of protective genes such as BDNF. Although existing evidence supports their potential as therapeutic targets, their complete upstream and downstream regulatory networks and tissue-specific functions still need further clarification.

##### 2.2.1.2 EZH2

EZH2 is the catalytic subunit of Polycomb Repressive Complex 2 (PRC2), which inhibits gene expression by catalyzing trimethylation of histone H3 lysine 27 (H3K27me3). In the rat middle cerebral artery occlusion/reperfusion (MCAO/R) model, EZH2 mediates histone H3K27me3, directly inhibiting the expression of the neuroprotective factor BDNF and aggravating brain injury [35]. Reducing H3K27me3 levels with its specific inhibitor GSK-126 can activate the PI3K/Akt/mTOR signaling pathway, thereby alleviating ischemia-hypoxia-induced neuronal apoptosis and neurological deficits [36, 37]. Additionally, SIRT1 can inhibit EZH2 activity through deacetylation modification, promote the expression of Forkhead box C1(FOXC1) and Ubiquilin 1(UBQLN1) to mitigate post-ischemic protein damage [38].

These results indicate that targeted inhibition of EZH2 exerts neuroprotective effects by promoting the expression of protective genes (such as BDNF), activating neural survival signals (such as PI3K/Akt), and maintaining intracellular protein homeostasis, making it a potential therapeutic target.

##### 2.2.1.3 SMYD2

SMYD2 (SET and MYND domain containing 2) belongs to the SMYD family. SMYD2 expression is significantly upregulated in cerebral microvascular endothelial cells after OGD/R treatment and in the ischemic penumbra of the mouse MCAO/R model. Knockdown of SMYD2 can reduce blood-brain barrier permeability and promote neurological function recovery, while overexpression of SMYD2 impairs endothelial cell integrity and aggravates blood-brain barrier disruption by activating the SphK/S1PR signaling axis [39].

##### 2.2.1.4 SETD7 and SETD1B

Both SETD7 and SETD1B belong to the SET domain-containing (SETD) family of methyltransferases. Studies have shown that targeted downregulation of SETD7 expression *via* miR-330-5p or miR-153-3p can significantly alleviate neuroinflammation and neuronal apoptosis [40, 41].

In the mouse MCAO/R model and OGD/R-treated HT22 cells, knockdown of SETD1B can reduce Transferrin Receptor 1 (TFR1) levels while upregulating the expression of Glutathione Peroxidase 4 (GPX4) and ferroportin (FPN), thereby improving the prognosis of ischemic stroke by inhibiting ferroptosis [42].

Therefore, inhibiting the abnormal expression of either SETD7 or SETD1B has clear neuroprotective potential. However, their precise upstream regulators, downstream targets, and cell type-specific functions still require in-depth research.

#### 2.2.2 Histone Demethylases

Histone demethylases mainly catalyze the removal of methyl groups from specific lysine residues of histone H3 (such as K4, K9, K27, K36) and are collectively referred to as lysine demethylases (KDMs). In recent years, multiple studies have revealed the important roles of various KDMs in the pathological process of ischemic stroke. The following focuses on KDMs associated with ischemic stroke in recent studies.

##### 2.2.2.1 KDM1A

Lysine-specific histone demethylase 1A (KDM1A) is the first identified histone demethylase, which can remove H3K4me1/me2 and H3K9me1/me2 modifications [43]. In the ischemic penumbra of mice after MCAO/R, KDM1A expression is significantly upregulated. Treatment with Artemisinin (ART) can effectively inhibit KDM1A expression and reduce ischemia-induced neuronal apoptosis. Further mechanistic studies have found that KDM1A can specifically remove the H3K4me2 modification in the promoter region of sphingosine kinase 2 (SPHK2), thereby inhibiting SPHK2 transcription. Overexpression of KDM1A or knockdown of SPHK2 can reverse the neuroprotective effect of ART [44].

##### 2.2.2.2 KDM2A

Lysine demethylase 2A (KDM2A) can specifically catalyze the removal of H3K36me2 modification [45]. In the rat Transient Global Cerebral Ischemia (TGCI) model and SH-SY5Y OGD/R model, KDM2A can promote β-Catenin protein degradation, increase post-ischemic neuronal apoptosis, and impair cognitive function. Knockdown of KDM2A can stabilize β-Catenin protein levels, thereby upregulating the expression of its downstream target gene survivin and exerting neuroprotective effects [46].

##### 2.2.2.3 KDM4A

Lysine demethylase 4A (KDM4A) can catalyze the removal of various repressive histone methylation modifications, including H3K9me2/3, H3K36me2/3, and H4K26me2/3 [47]. KDM4A expression is upregulated in the rat MCAO model, and inhibition of KDM4A can block the activation of the NF-κB pathway in microglia [48]. At the molecular level, KDM4A can remove the H3K9me3 modification in the promoter region of mouse double minute 2 homolog (MDM2), upregulate MDM2 expression, and thereby promote the ubiquitin-proteasomal degradation of downstream target complement-C1q/tumor necrosis factor-related protein 3(CTRP3). Inhibition or knockdown of KDM4A can reverse this process, inducing microglial polarization toward the M2 anti-inflammatory phenotype through the MDM2/ CTRP3 axis, ultimately reducing cerebral infarction [49].

##### 2.2.2.4 KDM6A

Histone lysine demethylase 6A (KDM6A) is a type of histone demethylase that can specifically remove H3K27me1/2/3 modifications [50]. KDM6A expression is significantly upregulated in mice after MCAO/R. Further studies have found that KDM6A can enhance the transcriptional activation of interferon regulatory factor 5 (IRF5) by removing the H3K27me3 modification at the IRF5 promoter, thereby aggravating the neuroinflammatory response after ischemia [51].

##### 2.2.2.5 KDM6B

Lysine demethylase 6B (KDM6B) can specifically remove H3K27me3 modification [52]. KDM6B expression is upregulated in the mouse MCAO/R model. KDM6B can directly bind to the promoter region of the bone morphogenetic protein 2 (BMP2) gene, promote BMP2 transcriptional activation by removing the H3K27me3 modification, and thereby upregulate the expression of BMP2 and its downstream target Bcl-2 modifying factor(BMF), aggravating cerebral infarction [53].

In summary, existing studies have found that KMTs exacerbate ischemic brain injury by specifically catalyzing the methylation of different lysine sites on histones (such as H3K9, H3K27), silencing neuroprotective genes, or activating damaging pathways. In contrast, KDMs exacerbate brain injury by removing methylation modifications at specific lysine sites on histones, abnormally activating damaging pathways, or silencing protective genes. The two act synergistically to aggravate brain injury through "methylation-mediated silencing of protective genes" and "demethylation-mediated activation of damaging pathways", respectively. Targeted inhibition of pro-damaging KMT/KDM isoforms and restoration of histone methylation balance have demonstrated clear neuroprotective effects in cellular and animal models. Future research needs to further clarify their upstream and downstream regulatory networks, cell type-specific functions, and tissue-selective roles, providing theoretical support for the development of highly specific epigenetic inhibitors and the advancement of precision therapy for ischemic stroke. The epigenetic regulatory mechanisms of different KMT and KDM isoforms in ischemic stroke are systematically summarized in Table **2**.

### 2.3 Histone Acetylation and Deacetylation

In eukaryotic cells, conserved lysine residues in the N-terminal tails of histones are core sites for acetylation modification. HATs use acetyl-CoA as a cofactor to transfer acetyl groups to the ε-amino groups of lysine residues, neutralizing the positive charge of histones and weakening their electrostatic binding to the DNA phosphate backbone. This leads to relaxation of nucleosome structure and chromatin opening, facilitating the binding of transcription factors and RNA polymerase to activate gene transcription. Conversely, HDACs can remove acetyl groups, resulting in histone deacetylation, stabilizing nucleosome structure, inducing chromatin condensation, and inhibiting the assembly of transcriptional complexes to silence gene expression [54]. The balance of activity between HATs and HDACs directly regulates histone acetylation levels and downstream gene transcriptional activity. The following section will focus on the mechanisms of action and regulatory value of HATs (with a focus on p300) and HDACs in ischemic stroke.

#### 2.3.1 Histone Acetyltransferases

##### 2.3.1.1 Histone Acetyltransferase p300

As a key transcriptional coactivator and chromatin modifier in the HAT family [55]. p300's acetyltransferase activity is involved in long-term potentiation and memory formation in the nervous system. Neuronal p300 expression decreases after cerebral ischemia, which is closely associated with cell apoptosis. In rat primary neurons treated with Oxygen-Glucose Deprivation (OGD), histone H3/H4 acetylation levels and CREB Binding Protein (CBP) expression decrease, and curcumin-induced inhibition of CBP activity exacerbates neuronal damage [56]. The transcription factor Myocardin-Related Transcription Factor A (MRTFA) exerts anti-apoptotic effects by upregulating BCL-2/MCL-1, and its activity is co-regulated by p300 and HDAC5. p300/MRTFA expression decreases in a hydrogen peroxide-induced neuronal apoptosis model, and overexpression of p300 reduces the apoptosis rate [57]. In addition, p300 and HDAC3 co-regulate MeCP2 lactylation. After ischemia, treatment with the HDAC3 inhibitor RGFP966 or the p300 activator CTB can promote this modification, inhibit the transcription of apoptosis-related genes such as PLA2G6 and PDCD4, and reduce infarct volume [58].

#### 2.3.2 Histone Deacetylases

The HDAC family plays a wide range of roles in physiological processes such as histone and non-histone modification, chromatin structure assembly, gene expression regulation, and cellular metabolism. Based on amino acid sequence homology and cofactor dependence, HDACs can be divided into four classes: Class I includes HDAC1, HDAC2, HDAC3, and HDAC8; Class II is further divided into subclass IIa (HDAC4, HDAC5, HDAC7, HDAC9) and subclass IIb (HDAC6, HDAC10); Class III includes sirtuin 1–7 (SIRT1–SIRT7, NAD⁺ dependent); Class IV includes HDAC11 [59].

##### 2.3.2.1 Class I Histone Deacetylases (HDACs)

Class I histone deacetylases are widely present in most cells and are mainly localized in the nucleus, exerting transcriptional repressive functions by catalyzing histone deacetylation [60]. After ischemic stroke, the expression and activity of Class I HDAC members such as HDAC1 and HDAC2 in the ischemic penumbra of brain tissue are significantly upregulated, and this increase persists from the acute phase to the recovery phase, exacerbating cerebral ischemia hypoxia injury [61-64].

###### 2.3.2.1.1 HDAC1 and HDAC2

HDAC1 and HDAC2 have distinct roles in ischemic stroke. In rat/mouse middle cerebral artery occlusion/ reperfusion (MCAO/R), photothrombotic stroke (PTS) models, and primary neuron OGD/R models, inhibiting ischemia-induced upregulation of HDAC1 can reduce acute phase inflammatory factor levels and alleviate ischemia-induced brain tissue damage by inhibiting p53 deacetylation [65-67].

Notably, in endothelin-1 (ET-1) induced cerebral ischemia models of Sprague Dawley (SD) rats and primary neuron OGD/R models, HDAC1 expression and activity in the ischemic penumbra decrease, and inhibiting HDAC1 exacerbates damage, while the HDAC1 activator (compound 5104434) can mitigate injury. This contradiction may be related to the model type and experimental time points [68, 69].

Increased HDAC2 expression after ischemia leads to decreased synaptic density and impaired plasticity [70]. In models such as MCAO/R and OGD/R, inhibiting HDAC2 activity or expression can reduce neuronal apoptosis by attenuating NF-κB pathway activation and inhibiting GSK3β expression [71-75].

###### 2.3.2.1.2 HDAC3

HDAC3 can modify histones and non-histones in neurons, astrocytes, and microglia, regulating myelin formation, neuronal differentiation, and plasticity [76]. Studies have confirmed that inhibiting HDAC3 expression/activity or knocking out microglial HDAC3 can alleviate post-ischemic microglia-mediated neuroinflammation, reduce cerebral infarct volume, decrease blood-brain barrier leakage, and promote neurological function recovery by regulating downstream factors such as Transcription Factor PU. 1(PU.1) and ZBP/p-IRF3 [77-84]. Specific knockout of microglial HDAC3 can also accelerate myelin debris clearance by recruiting macrophages, facilitating remyelination [85]. Clinical studies have shown that serum HDAC3 expression is elevated in patients with acute ischemic stroke and is positively correlated with disease severity [86].

##### 2.3.2.2 Class II Histone Deacetylases (HDACs)

Class II histone deacetylases (HDACs) are divided into two subclasses (IIa and IIb) based on differences in their catalytic domains. HDAC6, which is mainly localized in the cytoplasm, is the only exception; the other members of the Class II HDAC family are distributed in both the cytoplasm and the nucleus [87].

###### 2.3.2.2.1 HDAC4

HDAC4 is highly expressed in neurons and can shuttle between the nucleus and cytoplasm to regulate synaptic plasticity and memory-related transcriptional programs [88]. After ischemic stroke, nuclear translocation of HDAC4 occurs in neurons of the ischemic penumbra within 4 hours in the PTS model [89].

Its nuclear translocation function is phase-dependent: nuclear accumulation in the acute phase (day 3) of the MCAO/R model downregulates neuroprotective factors such as c-Fos and BCL-2, exacerbating injury, while paeoniflorin can improve prognosis by inhibiting nuclear translocation and promoting MEF2/BCL-2 expression [90, 91]. Nuclear translocation in the subacute and recovery phases (days 2, 7, 14) is associated with increased expression of neuronal plasticity markers such as microtubule-associated protein 2, which is beneficial for neural remodeling [92]. These findings suggest that HDAC4 may aggravate brain injury by repressing protective genes in the acute phase, yet promote neural network remodeling and improve neurological deficits during the subacute and recovery phases.

In terms of expression levels, high HDAC4 expression exerts a protective effect: overexpressing HDAC4 can reduce neuronal apoptosis by downregulating High Mobility Group Box-1 protein (HMGB1) [93]. Upregulation of HDAC4 by the Leonurus japonicus extract SCM-198 can inhibit MMP-9/NOX-4 to maintain blood-brain barrier integrity [94]. Clinical studies have shown that serum HDAC4 expression in patients with acute stroke is lower than that in the normal control group, and is negatively correlated with TNF-α, IL-1β levels, and National Institutes of Health Stroke Scale (NIHSS) scores [95].

In summary, HDAC4 is a potential therapeutic target for ischemic stroke: its nucleocytoplasmic shuttling exerts phase-dependent regulation of injury and repair, and upregulated expression exerts protective effects through anti-apoptosis and maintaining blood-brain barrier integrity. However, the intrinsic connection and regulatory mechanisms between these two processes still require in-depth research.

###### 2.3.2.2.2 HDAC9

HDAC9 expression is upregulated after ischemic stroke, exacerbating neuroinflammation by activating inflammatory pathways such as IκB/NF-κB and MAPKs [96-98]. Inhibiting or knocking out HDAC9 exerts neuroprotective effects: knocking down HDAC9 can reduce the levels of endoplasmic reticulum stress-related molecules such as Activating transcription factor 6(ATF6) and C/EBP-homologous protein(CHOP), and decrease stress-induced apoptosis [99, 100]. Silencing HDAC9 can increase GPX4 levels and inhibit neuronal ferroptosis [101]. The HDAC inhibitor BML210 upregulates cGKⅡ by blocking the HDAC9-MEF2 interaction, alleviating post-ischemic neuronal apoptosis [102].

###### 2.3.2.2.3 HDAC6

HDAC6 regulates neural cell survival, differentiation, and cognitive formation, and is crucial for nervous system development [103]. Under physiological conditions, HDAC6 is lowly expressed in the cerebral cortex. After ischemic stroke, its expression is significantly upregulated, and it is upregulated with nuclear translocation in neurons and astrocytes of the ischemic penumbra in the PTS model. Inhibiting HDAC6 activity in the acute phase through gene knockout or drugs such as HBOP and Tubastatin A can exert neuroprotective effects by promoting macrophage Migration Inhibitory Factor (MIF) acetylation [104], downregulating the NLRP3 inflammasome [105], and activating the Nrf2 pathway [106].

In summary, existing studies have shown that the pathological progression of HDACs in ischemic stroke presents a continuous process of "progressive injury aggravation, initiation of endogenous repair, consolidation of repair and remodeling", and the functions of various HDAC isoforms and p300 dynamically switch with the pathological phase. 1. Acute phase injury stage (0-24 hours after ischemia): Pro-injury HDAC isoforms dominate, and acetylation imbalance exacerbates cell death. This stage is mainly characterized by rapid downregulation of cerebral acetylation levels triggered by ischemic stress, explosive activation of pro-injury pathways, with HDAC1/2/6/9 as the main effector isoforms, and p300 function inhibited, collectively amplifying neuronal injury, apoptosis, inflammatory cascade reactions, and blood-brain barrier disruption. 2. Subacute phase injury containment stage (24 hours-7 days after ischemia): Repair-related HDAC isoforms are activated, and acetylation balance is initially restored. This stage is mainly characterized by "initiation of endogenous repair mechanisms, activation of pro-repair HDAC isoforms, and partial recovery of p300 activity". HDAC3/4 exert anti-injury effects, cooperating with p300 to contain injury spread and initiate preliminary repair. 3. Chronic phase repair and remodeling stage (7d after ischemia): Repair-dominant regulation, with acetylation balance driving functional recovery. This stage is mainly characterized by "attenuation of pro-injury HDAC isoform activity and continuous enhancement of repair-related pathways". HDAC3/4 functions persist, and the p300-dominated acetylation network drives neural remodeling, angiogenesis, and remyelination. Further improving the regulatory network and temporal characteristics of HDACs after ischemic stroke will help to precisely target various HDAC isoforms to improve neurological function recovery. The epigenetic regulatory mechanisms of major Class I and Class II HDACs in ischemic stroke are systematically summarized in Table **3**.

##### 2.3.2.3 Class III Histone Deacetylases (HDACs)

Sirtuins, a family of Class III histone deacetylases, can catalyze the deacetylation of histones and various non-histones, regulating key pathophysiological processes such as aging and cellular metabolism. This family consists of 7 members (SIRT1-SIRT7) with specific subcellular localizations: SIRT1, SIRT6, and SIRT7 are localized in the nucleus; SIRT2 is localized in the cytoplasm; and SIRT3, SIRT4, and SIRT5 are localized in mitochondria [107]. Under the pathological context of ischemic stroke, the functions of family members are bidirectional: SIRT1, SIRT3, SIRT6, and SIRT7 exert neuroprotective effects, while SIRT2 and SIRT5 exhibit neurotoxicity [108].

###### 2.3.2.3.1 SIRT1

In the central nervous system, SIRT1 is widely expressed in neurons, neural stem cells, neural precursor cells, astrocytes, and microglia [109]. Recent evidence indicates that SIRT1 plays important regulatory roles in multiple pathological processes after ischemic stroke [110].

*SIRT1 and Neuroinflammation*

Inflammation persists throughout the pathological process of stroke, and SIRT1 inhibits inflammatory responses through multiple pathways. Resveratrol can activate SIRT1 to mediate the deacetylation of the NF-κB p50 subunit, alleviating neuroinflammation, while the use of EX-527 (a SIRT1 inhibitor) exacerbates NF-κB activation and microglial polarization toward the proinflammatory phenotype [111, 112]. SIRT1 can also deacetylate HMGB1 to inhibit its activity and reduce inflammatory damage [113].

Additionally, SIRT1 and HDAC6 have a competitive relationship, co-regulating the expression of the NLRP3 inflammasome. Overexpression of SIRT1 reduces NLRP3 levels and antagonizes neuroinflammation, while inhibiting or knocking down SIRT1 enhances NLRP3 expression and aggravates brain injury [105, 114, 115].

*SIRT1 and Oxidative Stress*

Accumulation of Reactive Oxygen Species (ROS) after ischemic stroke induces cytotoxicity [116], and SIRT1 alleviates oxidative stress through dual mechanisms. On one hand, it activates the Nrf2 signaling pathway to upregulate the expression of antioxidant enzymes such as superoxide dismutase (SOD) and catalase (CAT) [117]. On the other hand, it enhances the transcriptional activity of FOXO (Forkhead box O, FOXO) family members through deacetylation, reducing oxidative damage after ischemia [118, 119].

*SIRT1 and Mitochondrial Dysfunction*

A disparity in mitochondrial fission and fusion triggers dysfunction and structural damage after an ischemic stroke. This compulsive change causes extreme accumulation, oxidative stress, and eventually synaptic death. It even leads to an inadequate mobile power supply. In ischemic stroke, the hallmark of cerebral death has been identified as nuclear damage [120].

After MCAO/R in mice, upregulating SIRT1 reduces mitochondrial ROS production and maintains the balance of mitochondrial fission and fusion [121]. Panax notoginseng leaf triterpenoids can upregulate PGC-1α through the SIRT1/PGC-1α pathway, enhancing mitochondrial energy metabolism and inhibiting injury [122]. Furthermore, upregulating SIRT1 expression can promote mitophagy through the SIRT1/PINK1/Parkin pathway to clear damaged mitochondria, and this protective effect depends on the synergistic interaction between SIRT1 and PINK1 [123].

*SIRT1 and Apoptosis*

SIRT1 reduces neuronal death through apoptosis-related pathways. Inhibiting SIRT1 increases the acetylation levels of p53 and NF-κB p65, promoting apoptosis [110, 124]. In contrast, upregulating SIRT1 exerts anti-apoptotic effects by upregulating the anti-apoptotic protein BCL-2 and downregulating the pro-apoptotic protein BAX [125-127].

*SIRT1 and the Blood-Brain Barrier*

SIRT1 maintains blood-brain barrier function through specific pathways. After Mesenchymal Stem Cells (MSCs) upregulate SIRT1, they can downregulate the levels of related molecules through the SIRT1/PKC/MMP-9/AQP4 pathway, reducing blood-brain barrier damage [121]. The natural compound quercetin can activate the SIRT1/Nrf2/HO-1 pathway, reducing ROS production to maintain blood-brain barrier permeability [128, 129].

*SIRT1 and Angiogenesis*

SIRT1 promotes angiogenesis and improves blood flow in the ischemic region by regulating VEGF-related pathways [130]. In the Oxygen-Glucose Deprivation/Reperfusion (OGD/R) model, upregulating SIRT1 can promote the proliferation and migration of cerebral microvascular endothelial cells through the SIRT1/HIF-1α/VEGF pathway [131]. Tetramethylpyrazine and panax notoginseng leaf triterpenoids upregulate VEGF expression through the SIRT1/ VEGFA and SIRT1/PGC-1α/ER pathways, respectively, promoting angiogenesis after ischemia [132, 133].

In summary, within the complex pathological network of ischemic stroke, SIRT1 forms a key regulatory network and exerts neuroprotective effects by coordinating multiple pathways. Studies have shown that SIRT1 significantly alleviates neuroinflammation by downregulating NLRP3 inflammasome expression, regulating the HMGB1/TLR4 axis, and inhibiting the NF-κB signaling pathway; it mitigates oxidative stress by activating the Nrf2/ARE and FOXO pathways; and improves mitochondrial function through PGC-1α. Additionally, SIRT1 maintains cellular homeostasis by promoting mitophagy, regulating p53 acetylation, and balancing Bcl-2/Bax. These mechanisms collectively constitute the multi-dimensional protective effects of SIRT1 in ischemic stroke, making it a highly promising therapeutic target.

###### 2.3.2.3.2 SIRT6

SIRT6 exhibits multiple neuroprotective effects in ischemic stroke, with its mechanisms involving key pathological processes such as angiogenesis, oxidative stress, neuroinflammation, and cell apoptosis.

Regarding angiogenesis, endothelial cell-specific SIRT6 knockout (eSIRT6⁻/⁻) in transient Middle Cerebral Artery Occlusion (tMCAO) mice exacerbates blood-brain barrier damage and increases endothelial cell Caspase-3 levels, while overexpression of SIRT6 reduces Caspase-3 and activates Akt to alleviate injury [134]. Egr2 (Early growth response protein 2) carried by bone marrow mesenchymal stem cell (BMSC)-derived exosomes can bind to the SIRT6 promoter to upregulate its expression, and SIRT6 inhibits the Notch1 signaling pathway through deacetylation, promoting eNOS/VEGF expression to enhance microvascular function [135].

In terms of regulating oxidative stress and neuroinflammation, overexpression of SIRT6 increases Nrf2 to counteract oxidative stress in MCAO/R models [136]. Upregulating SIRT6 inhibits microglial activation, reduces TXNIP to exert anti-inflammatory effects, and promotes angiogenesis [137].

Regarding neuronal ferroptosis and mitochondrial homeostasis, SIRT6 promotes Solute carrier family 7 member 11 (SLC7A11) expression through deacetylating FOXO2 or inhibiting the Nuclear receptor coactivator 4(NCOA4)- Ferritin heavy chain 1(FTH1) interaction, exerting anti-apoptotic and anti-ferroptotic effects [138, 139]. Activating the SIRT6/AMPK/PGC-1α/Akt axis promotes mitophagy [140].

Additionally, in clinical trials, detecting serum SIRT6 levels in patients with acute stroke revealed a significant negative correlation between SIRT6 levels and 90-day mortality. This suggests that SIRT6 expression may serve as a potential indicator for assessing disease prognosis, and its protective effects have clinical translational value.

###### 2.3.2.3.3 SIRT3

As the main mitochondrial deacetylase, SIRT3 has been shown to regulate pathophysiological processes in ischemic stroke through multiple signaling pathways.

SIRT3 can promote angiogenesis after ischemia. In the MCAO/R model, SIRT3 knockout mice exhibit reduced neurogenesis and angiogenesis, aggravated blood-brain barrier damage, and inflammation [141]. while overexpression of SIRT3 can alleviate barrier damage by inhibiting the HIF-1α pathway and regulating Vascular Endothelial Growth Factor (VEGF) [142]. Knocking down SIRT3 also inhibits mitophagy and angiogenesis [143].

SIRT3 maintains mitochondrial function and alleviates ischemic brain injury through multiple pathways. Inhibiting the opening of the mitochondrial permeability transition pore (MPTP). Overexpression of SIRT3 reduces MPTP-related proteins (VDAC1, ANT1) and Caspase-3, decreasing ROS release and apoptosis [144].

Activating the mitochondrial unfolded protein response (mtUPR), inducing mtUPR through the FOXO3/SphK1 pathway to clear misfolded proteins [145].

Regulating mitophagy and fusion: Stilbene glycoside enhances mitophagy through SIRT3/AMPK [146]. Overexpression of SIRT3 also increases OPA1 (Optic atrophy 1) activity, improving energy metabolism [147].

SIRT3 is involved in regulating inflammation, oxidative stress, and cell apoptosis. Enhancing SIRT3 activity inhibits NLRP3 inflammasome assembly, reducing inflammation and apoptosis [148]. It also antagonizes oxidative damage and cell death by inhibiting mitochondrial ROS production [149], promoting Nrf2-mediated antioxidant enzyme expression [150], enhancing BCL-2 function [151], and inhibiting necroptotic proteins such as PARP-1/AIF [152].

The epigenetic regulation mechanism of SIRT1, SIRT6, and SIRT3 in ischemic stroke has been systematically summarized in Table **4**.

##### 2.3.2.4 Other Sirtuin Proteins

###### 2.3.2.4.1 SIRT2

SIRT2 is mainly localized in the neuronal cytoplasm and exhibits clear neurotoxicity in ischemic stroke. SIRT2 expression in neurons of the ischemic region is significantly upregulated in tMCAO mice, and inhibiting SIRT2 with the specific inhibitor AGK2 or gene knockdown reduces cerebral infarct volume [153, 154]. Its mechanisms include inhibiting neuronal apoptosis by downregulating Forkhead box O3 (FOXO3a) [155] and inducing VEGF and Heme oxygenase 1(HO-1)expression to alleviate oxidative stress [156]. Clinical studies have shown that serum exosomal SIRT2 levels in patients with acute ischemic stroke are significantly higher than those in the control group [157]. This evidence from basic research to clinical observation shows that inhibiting the expression and activity of SIRT2 after ischemic stroke may be an effective treatment strategy to improve the prognosis.

###### 2.3.2.4.2 SIRT5

SIRT5 is a mitochondria-localized NAD⁺-dependent desuccinylase that plays a clear pathological role in ischemic stroke. SIRT5 levels are significantly increased in the blood of patients with acute ischemic stroke and the brain tissue of tMCAO mice, and SIRT5 knockout significantly reduces cerebral infarct volume in mice, confirming its negative regulatory role [158].

Mechanistically, SIRT5 can specifically mediate the desuccinylation of annexin A1 (ANXA1) at the K166 site, promoting excessive microglial activation and abnormal expression of proinflammatory factors, thereby exacerbating neuroinflammation; knocking down SIRT5 or intervening with the inhibitor MC3482 can effectively inhibit this inflammatory response [159, 160].

Clinical and animal studies have confirmed that inhibiting SIRT5 is a potential therapeutic strategy for ischemic stroke, but its regulatory network and interactions with other organelles still require in-depth exploration.

###### 2.3.2.4.3 SIRT4 and SIRT7

To date, no studies have directly investigated the roles of SIRT4 and SIRT7 in ischemic stroke. Whether they participate in regulating the pathophysiological mechanisms following ischemic stroke remains to be elucidated in future research.

### 2.4 Non-Coding RNAs and Ischemic Stroke

Non-coding RNAs (ncRNAs) are a class of functional RNA molecules that do not encode proteins. Based on functional characteristics and molecular length, they are mainly classified into three categories: housekeeping non-coding RNAs, small RNAs, and long non-coding RNAs (lncRNAs) [162]. Small RNAs are a class of non-coding RNAs with a length of approximately 16-200 nucleotides, among which microRNAs (miRNAs) are the most representative. They mainly function in the cytoplasm by binding to the 3' untranslated region (3' UTR) of target messenger RNAs (mRNAs), mediating mRNA degradation or translational inhibition, and participating in the regulation of gene expression in the nucleus. In addition, small RNAs include PIWI-interacting RNAs (piRNAs), which mainly achieve gene silencing through epigenetic and post-transcriptional regulatory mechanisms. Long non-coding RNAs refer to non-coding RNAs with a length exceeding 200 nucleotides. Both miRNAs and lncRNAs belong to regulatory non-coding RNAs and can participate in the regulation of gene expression at multiple levels, such as transcriptional, post-transcriptional, and epigenetic levels. Recent studies have shown that non-coding RNAs play important regulatory roles in the pathophysiological process of ischemic stroke (Fig. **2**). The following sections will focus on the latest research progress of miRNAs and lncRNAs in the field of ischemic stroke.

#### 2.4.1 miRNA

miRNAs are a class of highly conserved small non-coding RNAs with a length of approximately 20-40 nucleotides. In recent years, miRNAs have been extensively studied in the context of ischemic stroke [163]. Multiple clinical studies have compared serum samples from patients with acute ischemic stroke and healthy controls, and found a large number of miRNAs with significantly altered expression, including miR-34a [164], miR-181a [165], miR-338-3p [166], miR-1250-5p, and miR-3065-5p [167, 168]. These miRNAs are expected to become potential biomarkers for diagnosis and prognosis evaluation.

Animal studies (results of the past three years are summarized in Table **5**) have shown that miRNAs are involved in regulating various pathophysiological processes after ischemic stroke, such as excitotoxicity, neuroinflammation, oxidative stress, cell apoptosis, and angiogenesis. These findings indicate that miRNAs play key regulatory roles in different cell types, and targeted regulation of miRNA expression is a highly promising therapeutic strategy for intervening in ischemic stroke.

#### 2.4.2 lncRNA

In the early stage of lncRNA research, scholars found that many lncRNAs are localized in chromatin regions and can interact with chromatin modification complexes, suggesting that epigenetic regulation may be a key functional mechanism of lncRNAs. They can regulate DNA methylation status and affect histone modification patterns. With deeper research, it has been clarified that they can also regulate gene expression through multiple pathways, such as acting as competing endogenous RNAs (ceRNAs) to sponge miRNAs, cis/trans-regulating transcription, binding to RNA-binding proteins, and mediating chromatin remodeling [203, 204].

Similar to miRNAs, multiple clinical studies on Chinese populations have initially verified a set of lncRNAs with potential for diagnosis and prognosis evaluation. Among them, H19, GAS5, PVT1, TUG1, and MALAT1 have shown stable and consistent expression changes in independent cohorts, demonstrating good prospects as reliable biomarkers [205-208].

In addition, lncRNAs have been confirmed to be extensively involved in various pathophysiological processes after ischemic stroke (related regulatory mechanisms are summarized in Table **6**) [209-237]. Therefore, systematic and in-depth exploration of the mechanisms of action of lncRNAs in ischemic stroke is expected to identify valuable targets for the development of new therapeutic strategies.

## 3 APPLICATION OF EPIGENETIC THERAPY IN ISCHEMIC STROKE

The reversibility of epigenetic modifications at the DNA, RNA, and histone levels has made them a focus of research for the treatment of ischemic stroke. The core of epigenetic therapy is to reverse abnormal epigenetic states in pathological processes and restore the homeostasis of the brain epigenome. A variety of targeted drugs and non-coding RNAs have demonstrated efficacy in improving neurological function in animal models, laying the foundation for intervention. Although most central nervous system epigenetic drugs are still in the preclinical stage, breakthroughs and clinical successes in oncology have provided direction and precedents for stroke treatment [238].

### 3.1 DNMT Inhibitors (DNMTi)

DNA methyltransferase inhibitors (DNMTi) are the first epigenetic drugs clinically applied in cancer treatment, mainly classified into nucleoside analog inhibitors and non-nucleoside inhibitors [239, 240]. Among them, 5-azacytidine and 5-aza-2'-deoxycytidine are nucleoside DNMTi approved by the US FDA [241]. Both drugs have shown clear neuroprotective effects in various ischemic stroke models [3, 30, 242, 243]. Another nucleoside inhibitor, zebularine, is the first orally active DNMTi. Studies have shown that it can effectively reduce microglia-mediated neuroinflammation [243] and significantly reduce cerebral infarct volume in ischemic stroke models [244]. To avoid potential non-specific effects caused by the incorporation of nucleoside analogs into DNA/RNA, non-nucleoside DNMTi have been developed. RG108 is the first recognized small-molecule non-nucleoside DNMTi, which has shown potential in promoting the expression of axon-related genes and neural regeneration in neurological diseases such as post-stroke depression [245, 246]. Despite the demonstrated therapeutic potential of the above DNMTi in preclinical studies for ischemic stroke, their clinical translation faces multiple challenges: most studies on the protective effects of DNMT inhibitors in ischemic stroke remain at the cellular and short-term animal model stages, lacking preclinical translational research; in addition, potential off-target effects, central delivery issues, and insufficient understanding of the comprehensive and dynamic regulatory network of epigenetics limit the application of DNMTi in ischemic stroke. Future research needs to further clarify the precise map of DNA methylation at different stages after ischemia to determine optimal intervention targets and time windows, develop brain-targeted, lesion-specific novel inhibitors or delivery systems. Only by systematically addressing these limitations can DNA methylation regulation be truly transformed into a safe and effective therapeutic strategy for ischemic stroke.

### 3.2 TET Activation

L-ascorbic acid (vitamin C) has traditionally been considered to exert antioxidant effects after ischemic stroke [247]. Recent studies have shown that vitamin C can specifically bind to the C-terminal catalytic domain of TET enzymes, acting as a cofactor to indirectly activate TET enzymes, promote TET-mediated 5hmC formation, and thereby increase 5hmC levels [248]. In experimental models of ischemic stroke, vitamin C mediates TET3 activation to promote the transcriptional activation of various protective genes (especially anti-inflammatory genes), thereby reducing post-ischemic neuroinflammation and brain tissue damage [249].

In addition, other TET activators (such as mitoxantrone, flavonoids, and retinoic acid) have shown neuroprotective effects after ischemic stroke in multiple studies, including anti-apoptosis [250], anti-inflammatory [251], and antioxidant effects [252, 253], and maintenance of blood-brain barrier integrity [254]. However, the specific epigenetic mechanisms by which these compounds exert their effects in ischemic stroke still need to be fully elucidated.

### 3.3 MBD Protein Family and UHRF1

The regulation of DNA methylation extends far beyond interventions targeting "writers" (DNMTs) and "erasers" (TETs). Recent studies have shown that targeting "readers" can also correct abnormal methylation patterns under pathological conditions. In the mechanism of DNA methylation recognition, the MBD protein family and UHRF1 (containing PHD and RING finger domains) play core roles. MBD proteins can specifically recognize and bind to methylated CpG sites, establishing "silent chromatin" by recruiting co-repressor complexes containing HDACs, thereby serving as a bridge between DNA methylation and gene repression [255]. UHRF1 plays a key role in maintaining DNA methylation patterns; its SRA domain can recognize hemimethylated CpG sites, promote the base conversion of 5mC, and further recruit epigenetic regulators such as DNMT1, HDAC1, G9a, and SUV39H1 to enhance transcriptional repression [256].

In the field of cancer research, knocking down members of the MBD protein family or UHRF1 to reverse abnormal methylation patterns has shown significant anti-tumor effects [257]. In neuroscience research, investigators found that UHRF1 can regulate REST in a methylation-dependent manner, thereby promoting axon regeneration [258]. Clinical studies have shown that the mRNA levels of MBD proteins and UHRF1 in the brain tissue of patients with traumatic brain injury and Alzheimer's disease are significantly different from those in normal brain tissue [259]. In addition, studies have confirmed that the MeCP2 protein containing the MBD domain can enhance neural regeneration after ischemic stroke by promoting FoxO3 methylation and thereby upregulating zinc finger E-box binding homeobox 1(ZEB1 ) expression [260].

These findings indicate that the UHRF1 and MBD protein families may be involved in the regulation of the pathological process of ischemic stroke and are potential therapeutic targets. However, research on the specific functions and epigenetic mechanisms of these reader proteins in ischemic stroke is still insufficient. Future studies should focus on clarifying the mechanisms of action of the MBD protein family and UHRF1 in the pathophysiological process of ischemic stroke, with the aim of developing novel therapeutic strategies based on epigenetic reader proteins.

### 3.4 Histone Methyltransferase Inhibitors (EZH2 Inhibitors)

Significant progress has been made in the development of histone methyltransferase inhibitors in cancer treatment, especially EZH2 inhibitors. In ischemic stroke models, various EZH2 inhibitors have shown neuroprotective potential. Studies have confirmed that GSK126, a selective inhibitor of EZH2 enzymatic activity, can reduce neuronal apoptosis. Another inhibitor, DZNep, promotes microglial polarization toward the anti-inflammatory phenotype [261, 262]. These results indicate that both inhibiting EZH2 expression and blocking its enzymatic activity can exert neuroprotective effects after ischemic stroke.

### 3.5 Histone Deacetylase Inhibitors (HDACi)

The development of histone deacetylase inhibitors (HDACi) mainly focuses on Class I, IIa, and IIb HDACs. Based on chemical structure, these inhibitors can be divided into four categories, namely hydroxamic acids, short-chain fatty acids, cyclic tetrapeptides, and benzamides. These drugs reduce histone deacetylation levels in the promoter regions of target genes by inhibiting HDAC enzymatic activity or expression, thereby activating gene transcription. Building on their success in oncology, researchers are actively exploring the therapeutic potential of these drugs in ischemic stroke. A variety of HDAC inhibitors have shown therapeutic effects in ischemic stroke models. Among hydroxamic acid drugs, the non-selective inhibitor SAHA (suberoylanilide hydroxamic acid) can target HDAC1, 2, 3, 6, 7, and HDAC11, and has shown neuroprotective effects in various ischemic stroke models, including enhancing synaptic plasticity [71], inhibiting microglia-mediated neuroinflammation [263], and reducing blood-brain barrier damage [264]. Another hydroxamic acid inhibitor, TSA (trichostatin A), can specifically target HDAC1 and HDAC2, and studies have confirmed that it can promote neurological function remodeling after stroke [265, 266]. However, recent studies have shown that TSA may induce excessive neuronal autophagy by promoting H4K16 acetylation, thereby exacerbating brain damage, and its mechanism of action in ischemic stroke still needs further verification [267, 268].

The short-chain fatty acid HDAC inhibitor valproic acid can specifically inhibit HDAC1 and induce HDAC2 degradation. Studies have confirmed that this drug exerts anti-inflammatory and antioxidant effects by upregulating HSP70-1b, inhibiting glial scar formation during the recovery phase [269], preventing microglial polarization toward the proinflammatory phenotype [270], and reducing hippocampal neuronal apoptosis [271].

With the advancement of drug development, a variety of highly selective HDAC inhibitors have entered the preclinical research stage. Among them, the highly selective HDAC3 inhibitor RGFP966 has shown multiple protective effects in experimental ischemic stroke models [77, 80, 84, 272].

These results indicate that HDAC inhibitors have broad application prospects in the treatment of ischemic stroke. Future research should focus on developing more targeted HDAC inhibitors and further clarifying the optimal therapeutic time window, dose-response relationship, and long-term safety characteristics of different HDAC inhibitors.

### 3.6 Sirtuin Activation and Inhibition

Chemically synthesized SIRT1 activators (such as SRT2104 and SRT1720) reduce post-stroke neuroinflammation by inhibiting NF-κB activation in microglia [273, 274]. SRT2104 also provides dual anti-inflammatory and anti-apoptotic protection by reducing the acetylation levels of p53 and STAT3 [275]. Regarding SIRT6 activators, compounds such as MDL-811 and MDL-800 upregulate FOXC1 expression by promoting EZH2 deacetylation, thereby reducing neuroinflammation [276].

In addition to synthetic compounds, a variety of natural products also have the ability to activate Sirtuin family proteins: natural activators of SIRT1 include total saponins of Panax notoginseng [117], quercetin [277], forsythiaside B [278], and resveratrol [279]. Primary natural activators of SIRT6 are baicalein [138] and trilobatin [280]. Natural activators of SIRT3 include icariside II [281], luteolin [282], wogonoside [283], 4'-O-methylbavachalcone [152], trilobatin [284], and tetrahydroxystilbene glucoside [146]. These natural compounds have shown potential in improving neurological deficits after ischemic stroke by activating Sirtuins in experimental studies. However, since most natural products have multiple cardiovascular pharmacological activities in addition to regulating Sirtuins, whether their anti-ischemic stroke efficacy specifically depends on Sirtuin activation still needs further clarification.

Among SIRT2 inhibitors, AGK2 and AK-1 have been confirmed to inhibit neuronal apoptosis [154, 155]. Among SIRT2 inhibitors, AGK2 and AK-1 have been confirmed to inhibit neuronal apoptosis [285]. The specific SIRT5 inhibitor MC3482 reduces microglia-mediated neuroinflammation by blocking the desuccinylation of annexin A1 (ANXA1) [159].

These findings indicate that targeted inhibition of the abnormal activation of SIRT2 and SIRT5 after stroke is a highly promising therapeutic direction. Future research should focus on clarifying the optimal treatment timing, dose optimization, and long-term safety of these inhibitors to promote their clinical translation.

### 3.7 miRNA- and lncRNA-Based Therapies

Based on the above studies, delivering mimics or inhibitors of specific miRNAs or lncRNAs has become a highly promising strategy for the treatment of ischemic stroke. For example, in experimental ischemic stroke models, intracerebroventricular or intravenous injection of miR-210 inhibitors can significantly reduce the release of proinflammatory factors [286]. Conversely, administration of miR-181b-5p mimics can activate the Akt pathway and promote angiogenesis [170]. Similarly, treatment with DLX6-AS1 inhibitors encapsulated in lipid nanoparticles can effectively reduce neuroinflammatory responses [216].

### 3.8 Core Challenges in the Clinical Translation of Epigenetic Therapies for Ischemic Stroke

Currently, the clinical translation of stroke therapies based on epigenetic mechanisms faces four core obstacles. The primary challenge lies in the highly context-dependent nature of the target function. Key epigenetic regulators, such as DNMT3A and TET1, exhibit significant heterogeneity in their biological effects across different cell types and pathological stages [13, 14, 19, 20]. This characteristic implies that employing traditional global activation or inhibition strategies may lead to unpredictable consequences, potentially exacerbating neural damage. Therefore, developing technical tools capable of achieving cell-selective and spatiotemporal-specific interventions has become a paramount scientific issue in the field.

The second major obstacle stems from the limited efficiency of drug delivery to the central nervous system. While the blood-brain barrier maintains the homeostasis of the internal environment of the central nervous system, it severely restricts the ability of most therapeutic agents, particularly large-molecule nucleic acid drugs, to reach effective therapeutic concentrations at cerebral ischemic lesion sites. Constructing efficient, safe, and actively lesion-targeting delivery systems represents a critical bottleneck determining the successful translation of such therapies.

Third, the dynamic and complex nature of the therapeutic time window presents another formidable challenge. The pathological progression of ischemic stroke evolves rapidly over hours to weeks, and the functional state and interventional value of the same molecular target may differ fundamentally during the acute, subacute, and recovery phases [62, 92]. Precisely defining the optimal timing for different intervention targets and formulating individualized dynamic treatment plans based on this understanding is a critical challenge that must be addressed in translational research.

Finally, the potential off-target effects and long-term safety risks of epigenetic drugs cannot be overlooked. Many epigenetic modulators, such as broad-spectrum HDAC inhibitors and DNMT inhibitors, function by globally modifying the genome or chromatin. This mode of action may lead to unpredictable off-target effects and carries potential long-term risks, including carcinogenicity or interference with normal developmental processes [241, 246].

To address these systemic challenges, current research is advancing along three primary operational pathways:

(1)  Development of Brain-Targeted Precision Delivery and Smart Drug Technologies: Efforts are focused on creating novel brain-targeted delivery systems. These include lipid nanoparticles modified with specific ligands (*e.g*., targeting the transferrin receptor), engineered exosomes with natural biocompatibility and tissue homing capabilities, and physical methods such as focused ultrasound to achieve controlled, transient opening of the blood-brain barrier [287-290]. Concurrently, microenvironment-responsive prodrugs are being designed to activate only under pathological conditions specific to ischemic regions, such as low pH or high reactive oxygen species, thereby enhancing treatment precision and safety [291].
(2)  Construction of a Dynamic Biomarker Monitoring Network Based on Liquid Biopsy: There is an active pursuit to identify and validate stable epigenetic markers from peripheral blood, such as cell-free DNA methylation status of specific genes and exosomal non-coding RNA expression profiles [292]. The goal is to establish an objective indicator system capable of real-time assessment of a patient's pathological stage, prognostic prediction, and guidance for the timing of individualized treatment initiation, thereby advancing stroke therapy from an empirical model to a data-driven paradigm.
(3)  Exploration of Synergistic and Integrated Strategies for Epigenetic Therapies: Systematic evaluation is underway for combination regimens involving epigenetic drugs and existing standard therapies (*e.g*., intravenous thrombolysis, endovascular thrombectomy) or rehabilitation approaches [293]. This aims to investigate whether epigenetic interventions can effectively extend the neuroprotective time window, mitigate reperfusion injury, or enhance neuroplasticity induced by rehabilitation training, thereby establishing a new integrated therapeutic paradigm with synergistic effects.

## CONCLUSION

This review systematically elucidates the modular architecture of the epigenetic regulatory network following ischemic stroke. This network, operating through three regulatory tiers, DNA methylation, histone modifications, and non-coding RNAs, converges in a highly organized manner onto several core pathophysiological modules: inflammation and immune response, cell death, redox and metabolic homeostasis, and neurovascular unit repair. Different epigenetic factors achieve spatiotemporally specific and fine-tuned regulation of these core pathological processes by targeting specific nodes within the same modules. Various interventional strategies developed based on this understanding have demonstrated clear therapeutic potential in preclinical research.

Different epigenetic factors achieve spatiotemporally specific regulation of core pathological processes, such as neuroinflammation, cell death, oxidative stress, and neurovascular repair, by targeting distinct nodes within the same modules. Various interventional strategies developed based on this understanding have demonstrated clear therapeutic potential in preclinical research.

## FUTURE PERSPECTIVES

To overcome current obstacles in clinical translation and propel the field toward next-generation precision therapies, future research must seek breakthroughs in the following frontier directions:

(1)  Promoting a Paradigm Shift in Intervention: Exploring Locus-Specific Epigenetic Editing**.**

Epigenetic editing technologies based on the CRISPR/ dCas9 system, which fuse catalytically inactive dCas9 protein with epigenetic modifier enzymes, offer a revolutionary tool for achieving precise methylation or demethylation reprogramming at specific genomic loci [294]. This strategy has been conceptually established as a new paradigm for intervening in disease-related epigenetic states [295]. In the field of ischemic stroke, preclinical studies have preliminarily validated the feasibility of improving neurological outcomes by delivering a CRISPR/dCas9 activation system targeting the *SIRT1* gene [296].

(2)  Constructing a Temporally Precise and Individualized Therapeutic Network.

This involves two complementary directions. First, developing a new generation of epigenetic modulators with enhanced cell-type selectivity and spatiotemporal specificity [297, 298]. Second, deeply integrating liquid biopsy technology to provide an objective basis for determining the optimal individualized treatment timing by monitoring dynamic changes in patients' peripheral blood epigenetic profiles in real-time [299].

(3)  Innovating Intelligent Drug Delivery Systems.

Designing and optimizing intelligent delivery vectors capable of efficiently crossing the blood-brain barrier and actively targeting ischemic lesion cells is crucial for improving therapeutic efficacy and reducing systemic toxicity [300].

By deeply integrating these cutting-edge biotechnologies with practical clinical needs, epigenetic therapeutic strategies for ischemic stroke hold the promise of overcoming current bottlenecks and ultimately evolving into a new generation of precision treatment options capable of significantly improving patients' long-term prognoses.

## Figures and Tables

**Fig. (1):**
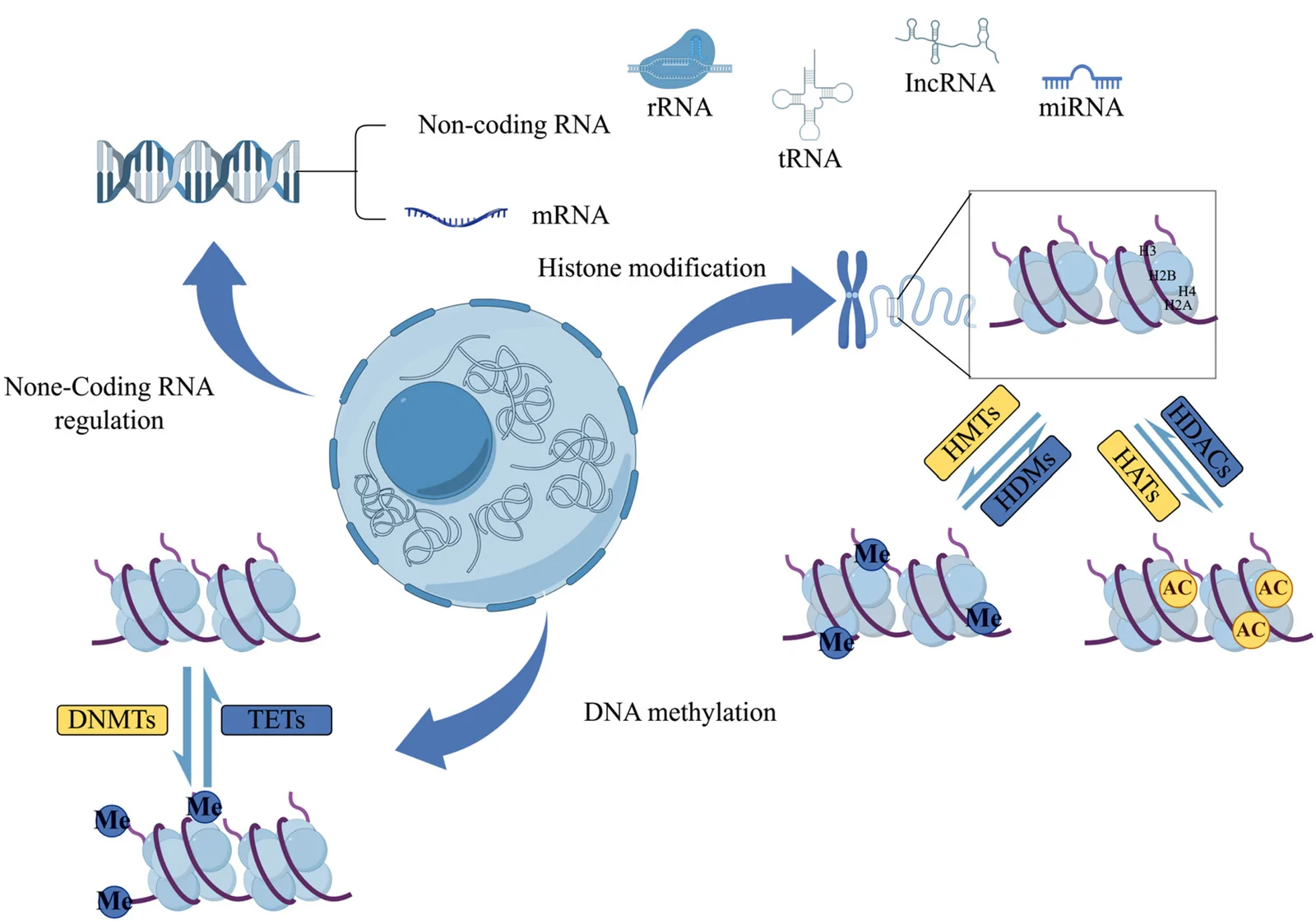
depicts fundamental epigenetic mechanisms, including DNA methylation, histone modifications, and non-coding RNA regulation, created using Figdraw 2.0 (https://www.figdraw.com).

**Fig. (2):**
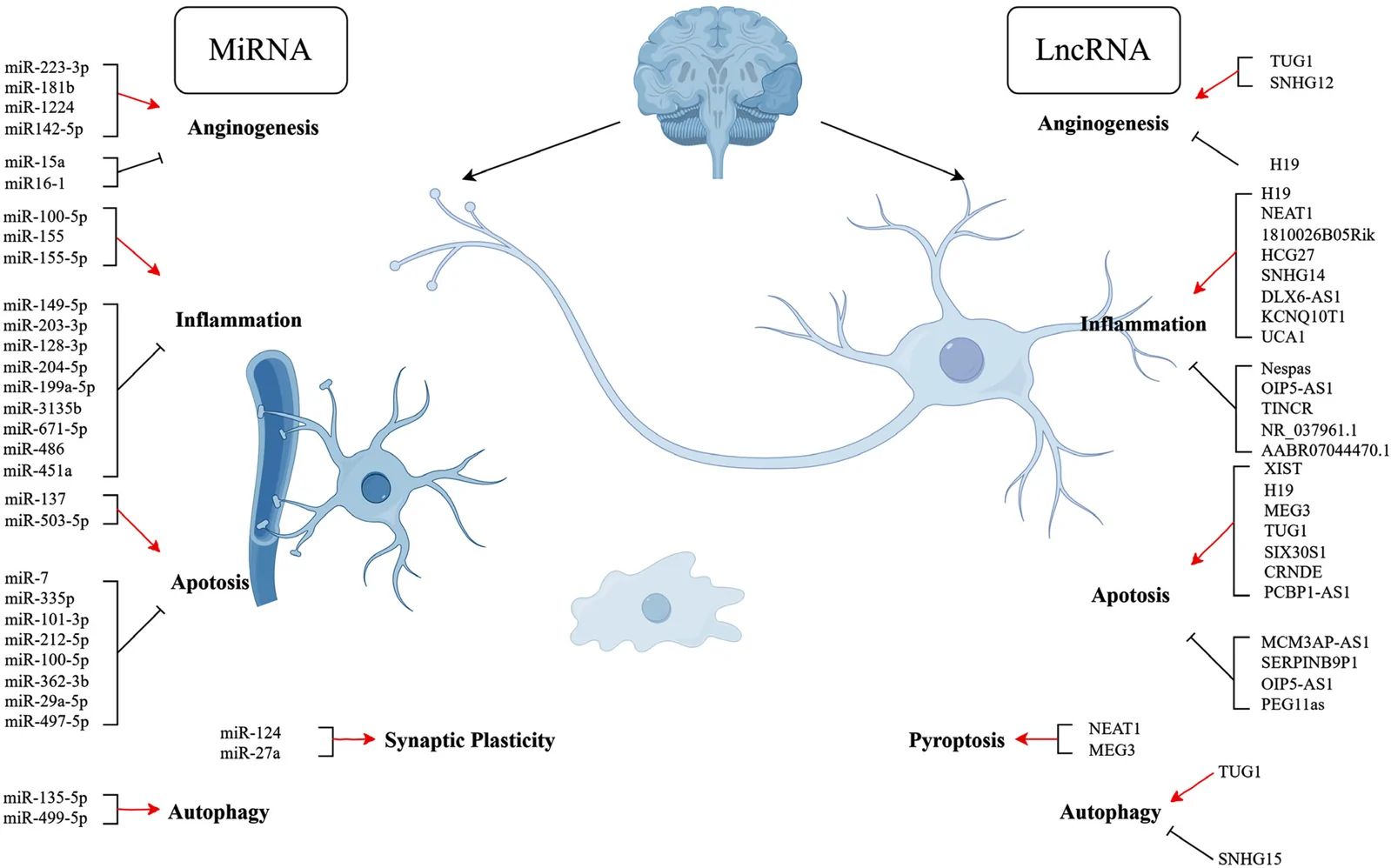
Roles of miRNA and lncRNA in pathophysiological processes after ischemic stroke. created using Figdraw 2.0 (https://www.figdraw.com).

**Table 1:** Epigenetic regulatory mechanisms of DNMT and TET isoforms in Ischemic stroke.

**Target**	**Study Model**	**Pathophysiological Mechanism**	**Regulatory Axis**	**Key Finding**	**References**
DNMT1	PTS Induced Mice	Apoptosis	/	DNMT1 inhibition reduces neuronal apoptosis induced by ischemia-hypoxia.	[30]
DNMT1	MCAO/R Induced Mice	BBB Integrity	DNMT1/TIMP-2/MMP-9	DNMT1 inhibition preserves blood-brain barrier integrity.	[10]
DNMT3A	MCAO/R Induced Mice	Inflammation	/	DNMT3A dysfunction post-stroke promotes pro-inflammatory pathway activation and neutrophil infiltration.	[14]
DNMT3A	MCAO/R Induced Rats	Mitophagy	DNMT3A/RHEBL-1	DNMT3A inhibition enhances Rhebl-1 expression, promotes mitophagy, and improves stroke outcome.	[13]
DNMT3A/3B	MCAO/R Induced Rats	Apoptosis, Oxidative Stress	DNMT3A,DNMT3B/PI3K/Akt	Inhibition or knockdown of DNMT3A/3B promotes PI3K demethylation, ameliorating stroke-induced apoptosis and oxidative stress.	[17]
DNMT3B	MCAO/R Induced Mice	Apoptosis	DNMT3B/MAP4K4	DNMT3B inhibition promotes MAP4K4 transcriptional activation and reduces neuronal apoptosis.	[16]
DNMT3B	MCAO/R Induced Rats	Ferroptosis	DNMT3B/PINK1	DNMT3B inhibition promotes PINK1 transcriptional activation and alleviates neuronal ferroptosis post-stroke.	[15]
TET1	MCAO/R Induced Rats	Apoptosis	TET1/ORP150	TET1 upregulates ORP150 expression, maintaining calcium homeostasis, preventing ER stress, and inhibiting apoptosis.	[19]
TET1	MCAO/R Induced Mice	Inflammation	TET1/RelB	TET1 reduces m⁵C modification of RelB mRNA, stabilizing its expression and exacerbating neuroinflammation.	[20]
TET2	MCAO/R Induced Mice	Synaptic Plasticity	TET2/BDNF	TET2 overexpression promotes BDNF transcription and improves stroke outcome.	[21]
TET2	MCAO/R Induced Mice	Inflammation	TET2/HDAC2/IL-6	TET2 recruits HDAC2, repressing IL-6 transcription *via* histone deacetylation.	[22]
TET2	MCAO/R Induced Mice	Inflammation	TET2/HDAC3/ NF-κB p65	TET2 recruits HDAC3, inhibiting NF-κB p65 acetylation and attenuating neuroinflammation.	[23]
TET3	MCAO/R Induced Mice	Neuroprotection	TET3/BDNF, VEGF, FOXO, Wnt	Post-stroke TET3 inhibition increases methylation of neuroprotective genes and exacerbates brain injury.	[26, 27]

**Table 2:** Epigenetic regulatory mechanisms of KMT and KDM isoforms in ischemic stroke.

**Target**	**Study Model**	**Pathophysiological Mechanism**	**Regulatory Axis**	**Key Finding**	**References**
G9a, SUV39H1	PTS Induced Mice	Apoptosis	G9a, SUV39H1/BDNF	Inhibition of G9a and SUV39H1 post-stroke promotes BDNF transcriptional activation.	[34]
EZH2	MCAO/R Induced Rats	Angiogenesis	EZH2/BDNF	EZH2 inhibition promotes BDNF transcriptional activation.	[35]
EZH2	MCAO/R Induced Rats	Apoptosis	EZH2/PI3K/Akt/mTOR	EZH2 inhibition reduces ischemia-induced apoptosis.	[37]
EZH2	MCAO/R Induced Rats	Proteostasis Disruption	EZH2/Foxc1/Ubqln1	Deacetylation of EZH2 promotes Foxc1 expression, subsequently upregulating Ubqln1 and reducing post-stroke protein damage.	[38]
SMYD2	MCAO/R Induced Mice	BBB Integrity	SMYD2/Sphk/S1PR	SMYD2 inhibition ameliorates blood-brain barrier damage after stroke.	[39]
SETD1B	MCAO/R Induced Mice	Ferroptosis	SETD1B/Tfrc	SETD1B inhibition reduces Tfrc expression and alleviates ferroptosis.	[42]
KDM4A	MCAO/R Induced Rats	Inflammation	KDM4A/NF-κB	KDM4A inhibition suppresses NF-κB activation and attenuates neuroinflammation.	[48]
KDM4A	MCAO/R Induced Mice	Inflammation	KDM4A/MDM2/CTRP3	KDM4A inhibition promotes microglial polarization toward an anti-inflammatory phenotype.	[49]
KDM6A	MCAO/R Induced Mice	Inflammation	KDM6A/IRF5	KDM6A inhibition suppresses IRF5 activation and alleviates neuroinflammation.	[51]
KDM6B	MCAO/R Induced Rats	Apoptosis	KDM6B/BMP2/Bmf	KDM6B inhibition reduces post-stroke apoptosis *via* Bmf downregulation.	[53]
KDM1A	MCAO/R Induced Mice	Apoptosis	KDM1A/SPHK2	KDM1A inhibition reduces stroke-induced apoptosis.	[44]
KDM2A	MCAO/R Induced Rats	Apoptosis	KDM2A/β-catenin	KDM2A inhibition stabilizes β-catenin protein and suppresses apoptosis.	[46]

**Table 3:** Epigenetic regulatory mechanisms of class I and class II HDAC isoforms in ischemic stroke.

**Target**	**Study Model**	**Pathophysiological Mechanism**	**Regulatory Axis**	**Key Finding**	**References**
HDAC3	MCAO/R Induced Mice	Inflammation	HDAC3/Nrf1/NF-κB p65	HDAC3 inhibition increases Nrf1 acetylation and suppresses p65, alleviating neuroinflammation	[77]
HDAC3	tFCI Induced Mice	Inflammation	HDAC3/PU.1	HDAC3 deficiency reduces PU.1-induced pro-inflammatory microglia without affecting anti-inflammatory microglial proliferation	[78]
HDAC3	MCAO/R Induced Mice	Inflammation	HDAC3/NF-κB p65/cGAS/STING	HDAC3 knockout suppresses p65 and enhances the cGAS/STING pathway, reducing neuroinflammation	[79]
HDAC3	MCAO/R Induced Mice	Inflammation	HDAC3/TLR4, HDAC3/Akt	HDAC3 inhibition reduces TLR4 and activates the Akt pathway, attenuating neuroinflammation and apoptosis	[80]
HDAC3	MCAO/R Induced Mice	Inflammation	HDAC3/ZBP1/p-IRF3	HDAC3 inhibition suppresses the interferon pathway and alleviates neuroinflammation	[81]
HDAC2	MCAO/R Induced Rats	Synaptic Plasticity	HDAC2/BDNF/TrkB	HDAC2 inhibition promotes BDNF expression and activates the TrkB pathway, enhancing synaptic plasticity	[71]
HDAC2	MCAO/R Induced Mice	Apoptosis	HDAC2/Reelin/GSK3β	HDAC2 inhibition suppresses GSK3β and reduces post-stroke neuronal apoptosis	[75]
HDAC9	MCAO/R Induced Rats	Inflammation	HDAC9/NF-κB	HDAC9 inhibition suppresses NF-κB activation and alleviates neuroinflammation	[96]
HDAC9	MCAO/R Induced Mice	Ferroptosis	HDAC9/HIF-1, Sp1/TRI, GPX4	HDAC9 inhibition reduces TRI, increases GPX4 expression, and alleviates ferroptosis	[101]
HDAC9	MCAO/R Induced Mice	Apoptosis	HDAC9/MEF2/cGKII	HDAC9 inhibition upregulates cGKII expression and reduces neuronal apoptosis	[102]
HDAC6	PTS Induced Mice	Apoptosis	HDAC6/MIF (K78)	HDAC6 inhibition promotes MIF acetylation at K78, reduces its nuclear translocation, and decreases neuronal apoptosis	[104]
HDAC6	MCAO/R Induced Mice	Inflammation	HDAC6/SIRT1/NLRP3	HDAC6 and SIRT1 antagonistically regulate NLRP3; HDAC6 inhibition suppresses NLRP3 and alleviates neuroinflammation	[105]
HDAC6	MCAO/R Induced Mice	Oxidative Stress	HDAC6/Nrf2/HO-1	HDAC6 inhibition promotes Nrf2 expression and alleviates oxidative stress injury	[106]

**Table 4:** Epigenetic regulatory mechanisms of SIRT1, SIRT6, and SIRT3 in ischemic stroke.

**Target**	**Study Model**	**Pathophysiological Mechanism**	**Regulatory Axis**	**Key Finding**	**References**
SIRT1	MCAO/R Induced Mice	Inflammation	SIRT1/NF-κB	SIRT1 activation post-stroke inhibits NF-κB, alleviating neuroinflammation.	[111]
SIRT1	OGD/R Induced N2a Cells	Inflammation	SIRT1/HMGB1/TLR4	SIRT1 activation promotes HMGB1 deacetylation, reducing neuroinflammation.	[113]
SIRT1	MCAO/R Induced Rats	Inflammation	SIRT1/NLRP3	SIRT1 activation inhibits NLRP3 inflammasome activation, mitigating neuroinflammation.	[105, 114]
SIRT1	MCAO/R Induced Rats	Oxidative Stress	SIRT1/Nrf2	SIRT1 activation promotes Nrf2 expression and upregulates antioxidant enzymes, reducing oxidative stress.	[117]
SIRT1	MCAO/R Induced Rats	Oxidative Stress	SIRT1/FOXO, PGC-1α	SIRT1 activation enhances FOXO and PGC-1α expression, attenuating oxidative stress damage.	[118]
SIRT1	MCAO/R Induced Rats	Mitochondrial Dysfunction	SIRT1/PGC-1α/OPA1, DRP1	SIRT1 activation promotes OPA1 and inhibits DRP1 expression, reducing mitochondrial fragmentation.	[161]
SIRT1	MCAO/R Induced Rats	Autophagy	SIRT1/PINK1/Parkin	SIRT1 activation triggers the PINK1/Parkin pathway, modulating autophagy and reducing brain injury.	[123]
SIRT1	MCAO/R Induced Mice	Apoptosis	SIRT1/BCL-2	SIRT1 activation upregulates BCL-2 expression, inhibiting neuronal apoptosis.	[126]
SIRT1	MCAO/R Induced Rats	BBB Integrity	SIRT1/PKCδ/MMP-9/AQP4	SIRT1 activation post-stroke inhibits MMP-9 and AQP4 expression, preserving BBB integrity.	[121]
SIRT1	MCAO/R Induced Rats	Angiogenesis	SIRT1/VEGFA	SIRT1 activation promotes VEGFA expression and enhances angiogenesis.	[132]
SIRT1	MCAO/R Induced Rats	Angiogenesis	SIRT1/PGC-1α/ERα	SIRT1 activation promotes angiogenesis *via* the ERα pathway.	[133]
SIRT6	MCAO/R Induced Mice	Angiogenesis	SIRT6/AKT, Caspase-3	SIRT6 knockout increases Caspase-3 and suppresses AKT activation, impairing vascular function.	[134]
SIRT6	MCAO/R Induced Mice	Angiogenesis	SIRT6/Notch1	SIRT6 activation promotes Notch1 deacetylation, inhibiting Notch signaling and improving cerebrovascular injury.	[135]
SIRT6	MCAO/R Induced Mice	Oxidative Stress	SIRT6/Nrf2	SIRT6 activation promotes Nrf2 expression, alleviating oxidative stress.	[136]
SIRT6	MCAO/R Induced Rats	Angiogenesis	SIRT6/TXNIP	SIRT6 upregulation inhibits microglial activation and TXNIP expression, promoting angiogenesis.	[137]
SIRT6	MCAO/R Induced Mice	Apoptosis	SIRT6/FOXO2/SLC7A11	SIRT6 upregulation promotes FOXO2 deacetylation and SLC7A11 expression, inhibiting apoptosis.	[138]
SIRT6	MCAO/R Induced Mice	Ferroptosis	SIRT6/NCOA4, FTH1	SIRT6 upregulation inhibits the NCOA4-FTH1 interaction, alleviating ferroptosis.	[139]
SIRT6	MCAO/R Induced Rats	Autophagy	SIRT6/AMPK/ PGC-1α/AKT	SIRT6 upregulation activates AKT and regulates mitophagy, reducing apoptosis.	[140]
SIRT3	MCAO/R Induced Mice	Angiogenesis	SIRT3/HIF-1α/VEGF	SIRT3 inhibits HIF-1α and modulates VEGF expression, promoting angiogenesis.	[142]
SIRT3	MCAO/R Induced Rats	Angiogenesis	SIRT3/PINK1/Parkin	SIRT3 activation promotes mitophagy and enhances angiogenesis.	[143]
SIRT3	MCAO/R Induced Mice	Mitochondrial Dysfunction	SIRT3/VDAC1, ANT1	SIRT3 activation inhibits VDAC1 and ANT1 expression, reducing mPTP over-opening.	[144]
SIRT3	MCAO/R Induced Mice	Mitochondrial Dysfunction	SIRT3/FOXO3/Sphk1	SIRT3 overexpression induces mtUPR, protecting neuronal mitochondria from damage.	[145]
SIRT3	OGD/R Induced PC12 Cells	Mitochondrial Dysfunction	SIRT3/AMPK	SIRT3 activation promotes neuronal mitophagy and inhibits apoptosis.	[146]
SIRT3	MCAO/R Induced Rats	Mitochondrial Dysfunction	SIRT3/OPA1	SIRT3 activation enhances OPA1 expression, inhibits apoptosis, and promotes mitochondrial biogenesis.	[152]
SIRT3	MCAO/R Induced Mice	Inflammation	SIRT3/NLRP3	SIRT3 activation inhibits NLRP3 inflammasome assembly, alleviating neuroinflammation.	[148]
SIRT3	MCAO/R Induced Mice	Apoptosis	SIRT3/BAX, BCL-2	SIRT3 activation promotes BCL-2 and suppresses BAX expression, inhibiting neuronal apoptosis.	[151]
SIRT3	MCAO/R Induced Rats	Apoptosis (Parthanatos)	SIRT3/PARP-1, PAR, AIF	SIRT3 activation reduces parthanatos-related proteins (PARP-1, PAR, AIF), inhibiting this death pathway.	[152]

**Table 5:** Epigenetic regulatory mechanisms of miRNAs in ischemic stroke.

**miRNA**	**Expression in IS**	**Target Gene**	**Pathophysiology Mechanism**	**Species**	**Axis**	**Conclusion**	**References**
miR-223-3p	Down	NOTCH2	Angiogenesis	OGD/R Induced HBMECs	miR-223-3p/ NOTCH2	Overexpression promotes post-stroke angiogenesis.	[169]
miR-181b	Down	PTEN	Angiogenesis	MCAO/R Induced Rats	miR-181b/ PTEN/Akt	Overexpression reduces infarct volume, promotes angiogenesis, and improves neurological function.	[170]
miR-1224	/	Serpine1	Angiogenesis	MCAO/R Induced Mice	miR-1224/ Serpine1	Upregulation promotes post-stroke angiogenesis and improves cerebral blood flow.	[171]
miR-142-5p	Down	ADAMTS1	Angiogenesis	MCAO/R Induced Rats	miR-142-5p/ ADAMTS1	Overexpression promotes eNOS release and contributes to angiogenesis in ischemic stroke rats.	[172]
miR-15a, miR-16-1	Up	Pappa2, Fgf9, Islr, Ccr2	Angiogenesis	MCAO/R Induced Mice	miR-15a, miR-16-1/ Pappa2, Fgf9, Islr, Ccr2	Knockout of miR-15a and miR-16-1 promotes post-ischemic angiogenesis.	[173, 174]
miR-149-5p	Down	Faslg, GRIN1	Inflammation, oxidative stress	MCAO/R Induced Rats	miR-149-5p/Faslg, GRIN1	Overexpression alleviates neuroinflammation, oxidative stress, and subsequent apoptosis post-stroke.	[175]
miR-203-3p	Down	Pde4d	Inflammation	MCAO/R Induced Mice	miR-203-3p/Pde4d	Overexpression reduces post-stroke inflammation and oxidative stress.	[176]
miR-128-3p	Down	/	Inflammation	MCAO/R Induced Mice	/	Upregulation inhibits microglial activation and alleviates neuroinflammation post-stroke.	[177]
miR-204-5p	Down	EphA4	Inflammation, apoptosis	MCAO/R Induced Rats	miR-204-5p/ EphA4/PI3K/AKT	Overexpression alleviates neuroinflammation and apoptosis post-stroke.	[178]
miR-199a-5p	Down	PIK3R2	Inflammation	MCAO/R Induced Rats	miR-199a-5p/ PIK3R2/AKT	Overexpression inhibits post-stroke neuroinflammation and maintains blood-brain barrier integrity.	[179]
miR-3135b	/	NF-κB	Inflammation	MCAO/R Induced Mice	miR-3135b/ NF-κB/IKKβ	Overexpression alleviates the post-stroke inflammatory response.	[180]
miR-100-5p	Up	TLR7	Inflammation	MCAO/R Induced Mice	miR-100-5p/ TLR7/NF-κB	Knockdown reduces post-stroke neuroinflammation.	[181]
miR-671-5p	Down	NF-κB	Inflammation	MCAO/R Induced Mice	miR-671-5p/ NF-κB/MMP-9	Overexpression inhibits post-stroke inflammation and maintains blood-brain barrier integrity.	[182]
miR-486	/	PTEN	Inflammation	MCAO/R Induced Rats	miR-486/PTEN	Promotes microglial polarization toward an anti-inflammatory phenotype and enhances neurogenesis.	[183]
miR-155	Up	Wnt	Inflammation	MCAO/R Induced Mice	miR-155/Wnt/ β-catenin	Inhibition of miR-155 expression alleviates post-ischemic neuroinflammation.	[184]
miR-155-5p	Up	IRF2BP2	Inflammation, oxidative stress	OGD/R Induced HMC3, SH-SY5Y	miR-155-5p/ IRF2BP2	Inhibition alleviates OGD/R-induced oxidative stress and inflammatory response.	[185]
miR-451a	Up	Akt	Inflammation	MCAO/R Induced Mice	miR-451a/ Akt/mTOR	Mimics suppress NK cell cytotoxicity and alleviate inflammation.	[186]
miR-7	Down	α-syn	Apotosis	MCAO/R Induced Mice	miR-7/α-syn	Supplementation increases α-syn expression and promotes neurological recovery.	[187]
miR-335-5p	/	IRAK1	Apotosis	MCAO/R Induced Rats	miR-335-5p/ IRAK1	Overexpression alleviates neuronal damage post-stroke.	[188]
miR-101-3p	Down	HDAC9	Apotosis	MCAO/R Induced Mice	miR-101-3p/ HDAC9	Overexpression alleviates post-stroke neuronal apoptosis.	[189]
miR-212-5p	Down	PLXNA2	Apotosis	MCAO/R Induced Rats	miR-212-5p/ PLXNA2	Overexpression effectively reduces infarct volume and restores motor function in MCAO/R rats.	[190]
miR-100-5p	/	NOX4	Apotosis	MCAO/R Induced Mice	miR-100-5p/NOX4	Administration of exogenous miR-100-5p effectively reduces cerebral infarct volume and neuronal apoptosis.	[191]
miR-362-3p	/	MCAM	Apotosis	MCAO/R Induced Rats	miR-362-3p/ MCAM/Wnt/ β-catenin	Exogenous increase reduces apoptosis of endogenous neural stem cells.	[192]
miR-29a-5p	Down	GSK-3β, AQP4	Apotosis	MCAO/R Induced Rats	miR-29a-5p/GSK-3β, AQP4	Upregulation alleviates post-ischemic blood-brain barrier disruption and reduces cerebral infarct volume.	[193]
miR-497-5p	Down	BDNF	Apotosis	OGD/R Induced PC12	miR-497-5p/BDNF/ TrkB/Akt	Overexpression suppresses BDNF expression and exacerbates brain injury post-stroke.	[194]
miR-137	Up	Sirt1	Apotosis	MCAO/R Induced Mice	miR-137/Sirt1	Silencing alleviates post-stroke neuronal apoptosis.	[195]
miR-503-5p	Up	USP10	Apotosis	MCAO/R Induced Mice	MiR-503-5p/USP10	Upregulation inhibits USP10 expression, promotes endoplasmic reticulum stress, and exacerbates neuronal apoptosis.	[196]
miR-146a-5p	Up	Eif4g2	Endoplasmic Reticulum Stress	MCAO/R Induced Rats	miR-146a-5p/ Eif4g2	Inhibition reduces endoplasmic reticulum stress-induced neuronal injury.	[197]
miR-135a-5p	Down	mTOR	Autophagy	MCAO/R Induced Rats	miR-135a-5p/ mTOR/NLRP3	Upregulation inhibits post-stroke autophagy and alleviates cognitive dysfunction.	[198]
miR-499-5p	/	PDCD4	Autophagy	HT22	miR-499-5p/ PDCD4/ATG5	Overexpression promotes neuronal autophagy, thereby alleviating stroke-induced neuronal damage.	[199]
miR-124	/	Dll4, AAK1	Synaptic plasticity	MCAO/R Induced Mice	miR-124/Dll4, Notch1; miR-124/AAK1/Notch	Overexpression induces astrocyte-to-neuron conversion and promotes functional recovery post-stroke.	[200, 201]
miR-27a	Up	SEMA6A	Synaptic plasticity	MCAO/R Induced Mice	miR-27a/ SEMA6A	Overexpression promotes axonal remodeling and improves neurological outcomes.	[202]

**Table 6:** Epigenetic regulatory mechanisms of LncRNAs in ischemic stroke.

**LncRNA**	**Expression in IS**	**Target Gene**	**Pathophysiology Mechanism**	**Species**	**Axis**	**Conclusion**	**References**
H19	Up	/	Inflammation	MCAO/R Induced Rats	/	Knockdown of H19 inhibits neuroinflammation post-ischemic stroke.	[209]
Nespas	Up	Shp2	Inflammation	MCAO/R Induced Rats	Nespas/miR-383-3p/ Shp2/NLRP3	Upregulation of Nespas effectively inhibits NLRP3 inflammasome activation post-stroke.	[210]
NEAT1	Up	rac1	Inflammation	MCAO/R Induced Mice	NEAT1/miR-488-3p/ rac1	Knockdown of NEAT1 inhibits inflammation and reduces astrocyte activation induced by MCAO.	[211]
OIP5-AS1	Down	irf2bp2	Inflammation, Oxidative stress	OGD/R Induced SH-SY5Y HMC3	OIP5-AS1/ miR-155-5p/ IRF2BP2	Overexpression of OIP5-AS1 sponges miR-155-5p, promotes IRF2BP2 expression, and inhibits post-stroke inflammation and oxidative stress.	[185]
1810026B05Rik	Up	NF-κB	Inflammation	MCAO/R Induced Mice	1810026B05Rik/ IκBα/NF-κB	Knockdown of 1810026B05Rik alleviates neuroinflammation after stroke.	[212]
HCG27	Up	miR-27a-3p	Inflammation	MCAO/R Induced Rats	HCG27/miR-27a-3p	Silencing HCG27 alleviates post-stroke inflammation and improves cognitive impairment in rats.	[213]
SNHG14	Up	EZH2	Inflammation	MCAO/R Induced Rats	SNHG14/EZH2/ BDNF	Downregulation of SNHG14 reduces inflammatory factors and apoptosis, and increases microvascular density.	[35]
TINCR	Down	miR-125b-5p	Inflammation	MCAO/R Induced Rats	TINCR/miR-125b-5p	Overexpression of TINCR inhibits post-stroke inflammatory response and neuronal apoptosis.	[214]
NR_037961.1	Down	SOCS3	Inflammation	MCAO/R Induced Mice	NR_037961.1/ SOCS3/JAK2/STAT3	Overexpression of NR_037961.1 promotes microglial polarization toward an anti-inflammatory phenotype post-stroke.	[215]
DLX6-AS1	Up	Nrf2	Inflammation	MCAO/R Induced Mice	DLX6-AS1/Nrf2/ HO-1/NLRP3	Knockdown of DLX6-AS1 reduces the release of inflammatory factors post-stroke.	[216]
AABR07044470.1	Down	PERM1	Inflammation	MCAO/R Induced Mice	AABR07044470.1/ miR-214-3p/PERM1	Overexpression of AABR07044470.1 significantly reduces NLRP3 inflammasome activation and inflammatory factor release.	[217]
KCNQ1OT1	Up	PTBP1	Inflammation	MCAO/R Induced Mice	KCNQ1OT1/ miR-16-5p/ PTBP1/SIRT1	Silencing KCNQ1OT1 reduces the production of pro-inflammatory factors post-stroke.	[218]
UCA1	Up	miR-18a-5p	Inflammation	MCAO/R Induced Rats	UCA1/miR-18a-5p	Knockdown of UCA1 reduces OGD/R-induced inflammatory factors.	[219]
XIST	Up	TRAF3	Apoptosis and Inflammation	OGD/R Induced SH-SY5Y	XIST/miR-25-3p/ TRAF3	Silencing XIST reduces apoptosis and inflammation after OGD/R.	[220]
H19	Up	IGF1R	Apotosis	MCAO/R Induced Mice	H19/let-7a/IGF1R	H19 silencing significantly reduces cerebral infarct volume after MCAO/R.	[221]
MCM3AP-AS1	Down	miR-27b-3p	Apotosis	MCAO/R Induced Mice	MCM3AP-AS1/ miR-27b-3p	Upregulation of MCM3AP-AS1 reduces infarct volume and improves cognitive dysfunction in MCAO/R mice.	[222]
MEG3	Up	NLRP3	Apotosis	MCAO/R Induced Mice	MEG3/miR-145-5p/ TLR4/NLRP3	Knockdown of MEG3 reduces neuronal apoptosis and decreases cerebral infarct volume.	[223]
SERPINB9P1	Down	HSPA2	Apotosis	OGD/R Induced A172	SERPINB9P1/ HSPA2	Overexpression of SERPINB9P1 inhibits OGD/R-induced inflammation and apoptosis.	[224]
OIP5-AS1	Down	TAB2	Apotosis	MCAO/R Induced Mice	OIP5-AS1/TAB2	Overexpression of OIP5-AS1 inhibits neuronal apoptosis.	[225]
TUG1	Up	HuR	Apotosis	MCAO/R Induced Rats	TUG1/HuR	Knockdown of TUG1 reduces neuronal apoptosis after ischemia.	[226]
SIX3OS1	Up	miR-511-3p	Apotosis	MCAO/R Induced Rats	SIX3OS1/ miR-511-3p	Silencing SIX3OS1 alleviates MCAO/ R-induced neuroinflammation and apoptosis.	[227]
OIP5-AS1	Down	TXNIP	Apotosis	MCAO/R Induced Mice	OIP5-AS1/TXNIP	OIP5-AS1 promotes TXNIP ubiquitination and degradation, reducing neuronal apoptosis post-stroke.	[228]
CRNDE	Up	miR-489-3p	Apotosis	OGD/R Induced SH-SY5Y	CRNDE/miR-489-3p/ FOXO3	Knockdown of CRNDE alleviates inflammatory factor release and reduces neuronal apoptosis after OGD/R.	[229]
PCBP1-AS1	Up	miR-506-3p	Apotosis	OGD/R Induced SH-SY5Y	PCBP1-AS1/miR-506-3p/CCL2	Silencing PCBP1-AS1 reduces neuronal apoptosis after OGD/R.	[230]
PEG11as	Up	miR-342-5p	Apoptosis	MCAO/R Induced Mice	PEG11as/ miR-342-5p/PFN1	PEG11as binds to miR-342-5p, promoting PFN1 expression and exacerbating neuronal apoptosis post-stroke.	[231]
H19	Up	IMP2	Angiogenesis	MCAO/R Induced Rats	H19/IMP2	Silencing H19 enhances IMP2 expression, promotes angiogenesis, and alleviates BBB injury.	[232]
TUG1	Up	HuR	Angiogenesis	MCAO/R Induced Mice	TUG1/HuR/VEGFA	TUG1 promotes HuR ubiquitination, reduces VEGFA stability, and inhibits angiogenesis.	[233]
NEAT1	Up	nlrc4	pyroptosis	MCAO/R Induced Rats	NEAT1/NLRC4	Inhibition of NEAT1 suppresses NLRC4 inflammasome activation and reduces neuronal pyroptosis post-stroke.	[234]
MEG3	Up	NLRP3	pyroptosis	MCAO/R Induced Rats	MEG3/NLRP3/ Caspase-1/GSDMD	Knockdown of MEG3 inhibits NLRP3 activation, reduces inflammatory factor release, and suppresses pyroptosis.	[235]
TUG1	Up	PINK1	Autophagy	PTS Induced Mice	TUG1/PINK1/Parkin	Knockdown of TUG1 enhances mitophagy and alleviates neuronal injury post-stroke.	[236]
SNHG15	Up	miR-153-3p	Autophagy	MCAO/R Induced Mice	SNHG15/ miR-153-3p/ATG5	Downregulation of SNHG15 inhibits neuronal autophagy and reduces apoptosis post-stroke.	[237]

## LIST OF ABBREVIATIONS

5caC: 5-carboxylcytosine
5fC: 5-formylcytosine
5hmC: 5-hydroxymethylcytosine
5mC: 5-methylcytosine
AAK1: Adaptor-associated Kinase 1
AIF: Apoptosis-inducing Factor
AKT: Protein kinase B
AMPK: AMP-activated Protein Kinase
ANXA1: Annexin A1
AQP4: Aquaporin 4
ART: Artemisinin
ATF6: Activating Transcription Factor 6
ATG5: Autophagy-related 5
BAX: Bcl-2-associated X Protein
BCL-2: B-cell Lymphoma 2
BDNF: Brain-derived Neurotrophic Factor
BMF: Bcl-2 Modifying Factor
BMP2: Bone Morphogenetic Protein 2
BMSC: Bone Marrow Mesenchymal Stem Cell
Caspase-3: Cysteine-aspartic Acid Protease 3
CBP: CREB-binding Protein
ceRNA: Competing Endogenous RNA
CHOP: C/EBP-homologous Protein
CpG: Cytosine-phosphate-guanine
CTRP3: Complement-C1q/tumor Necrosis Factor-Related Protein 3
DNMT: DNA Methyltransferase
DNMTi: DNMT Inhibitor
EZH2: Enhancer of Zeste Homolog 2
FBP1: Fructose-1,6-bisphosphatase 1
FOXO: Forkhead Box O
GPX4: Glutathione Peroxidase 4
GSK3β: Glycogen Synthase Kinase 3 beta
HAT: Histone Acetyltransferase
HDAC: Histone Deacetylase
HDACi: HDAC Inhibitor
HDM: Histone Demethylase
HIF-1α: Hypoxia-inducible Factor-1 alpha
HMGB1: High mobility Group Box 1 Protein
HMT: Histone Methyltransferase
HO-1: Heme Oxygenase 1
IL-6: Interleukin 6
IRF5: Interferon Regulatory Factor 5
KDM: Lysine Demethylase
lncRNA: Long Non-coding RNA
MAP4K4: Mitogen-activated Protein Kinase Kinase Kinase Kinase 4
MBD: Methyl-CpG Binding Domain
MBD2: Methyl-CpG Binding Domain Protein 2
MCAO/R: Middle Cerebral Artery Occlusion/Reperfusion
MDM2: Mouse Double Minute 2 Homolog
MEF2: Myocyte Enhancer Factor 2
miRNA: MicroRNA
MMP-9: Matrix Metalloproteinase 9
MPTP: Mitochondrial Permeability Transition Pore
mTOR: Mammalian Target of Rapamycin
NCOA4: Nuclear Receptor Coactivator 4
NCX1: Sodium-calcium Exchanger 1
NF-κB: Nuclear FACTOR KAPPA B
NIHSS: National Institutes of Health Stroke Scale
NLRP3: NLR Family Pyrin Domain Containing 3
Nrf2: Nuclear Factor Erythroid 2-related Factor 2
OGD/R: Oxygen-glucose Deprivation/Reoxygenation
ORP150: Oxygen-regulated Protein 150
p300: Histone Acetyltransferase p300
PARP-1: Poly(ADP-ribose) Polymerase 1
PDCD4: Programmed Cell Death Protein 4
PGC-1α: Peroxisome Proliferator-activated Receptor Gamma Coactivator 1-alpha
PI3K: Phosphatidylinositol 3-kinase
PINK1: PTEN-induced Putative Kinase 1
PTS: Photothrombotic Stroke
REST: Repressor Element-1 Silencing Transcription Factor
ROS: Reactive Oxygen Species
SIRT: Sirtuin
SMYD2: SET and MYND Domain Containing 2
SPHK2: Sphingosine Kinase 2
STAT3: Signal Transducer and Activator of Transcription 3
SUV39H1: Suppressor of Variegation 3-9 Homolog 1
TAB2: TGF-beta Activated Kinase 1/MAP3K7 binding protein 2
TDG: Thymine DNA Glycosylase
TET: Ten-eleven Translocation
TFR1: Transferrin Receptor 1
TGF-β: Transforming Growth Factor beta
TIMP2: Tissue Inhibitor of Metalloproteinases 2
TIR: TIR Domain Adaptor Protein
TLR4: Toll-like Receptor 4
TNF-α: Tumor Necrosis Factor Alpha
TXNIP: Thioredoxin-interacting Protein
UHRF1: Ubiquitin-like with PHD and RING finger domains 1
VEGF: Vascular Endothelial Growth Factor
ZEB1: Zinc Finger E-box-binding Homeobox 1
